# New components in genetic regulation of age‐dependent compound leaf architecture in pea

**DOI:** 10.1111/nph.71515

**Published:** 2026-08-14

**Authors:** Jacqueline K. Vander Schoor, Chantelle J. Beagley, Warwick Gill, Grégoire Aubert, Judith Burstin, Robert J. E. Wiltshire, Valérie Hecht, James L. Weller

**Affiliations:** ^1^ School of Natural Sciences University of Tasmania Hobart TAS 7001 Australia; ^2^ ARC Centre of Excellence for Plant Success in Nature and Agriculture University of Tasmania Hobart TAS 7001 Australia; ^3^ Tasmanian Institute of Agriculture (TIA) University of Tasmania Hobart TAS 7001 Australia; ^4^ INRAE, UMR Agroécologie Université Bourgogne Europe Dijon 21000 France

**Keywords:** compound leaf development, *CURLY LEAF*, flowering time, heteroblasty, legume, *PICKLE*, *Pisum sativum*, *SEUSS*

## Abstract

- Pea (*Pisum sativum* L.) is a classic model for compound leaf development. Pea leaves typically consist of a proximal series of paired lateral leaflets substituted distally by filiform tendrils, and the number of lateral organ pairs increases with ontogeny. This gradient has been proposed as an expression of heteroblasty, but no clear links with known leaf‐development pathways have been demonstrated to date.
- We explored potential links between lateral organ number, heteroblasty and flowering time in pea using natural and induced genetic variation, and characterize four distinct loci regulating compound leaf architecture through genetic and molecular analyses.
- Two previously described *AEROMACULATA* (*AERO*) loci *AERO1* and *AERO2* are identified as putative orthologs of *Arabidopsis* chromatin modifiers *CURLY LEAF* and *PICKLE*, and two novel loci *EXTRA PINNA PAIRS 1* (*EPP1*) and *EPP2* as co‐orthologs of the transcriptional adaptor *SEUSS*. These loci interact to influence a range of pleiotropic phenotypes related to meristem function.
- *AERO* and *EPP* genes controlling lateral organ number in pea leaves have no clear association with leaf complexity or heteroblasty in other systems, and represent new components in regulation of legume compound leaf development.

## Graphical abstract

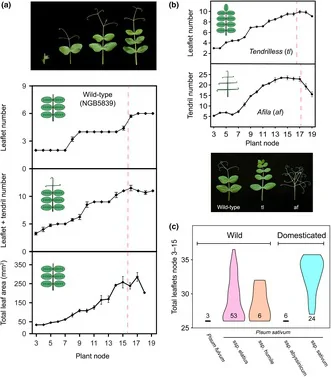

## Introduction

Leaf primordia arise at the periphery of the shoot apical meristem (SAM) and develop the characteristic shape, size and anatomical complexity of a mature leaf through cell division, growth and differentiation along multiple axes (Satterlee & Scanlon, 2019). At the molecular level, leaf growth and development are mediated through complex networks that incorporate hormones, sugars and regulators of gene expression, and the remarkable natural diversity in leaf form is largely generated through species‐specific modifications in these basic developmental processes and programs (Nakayama *et al*., 2022). In addition, even within an individual plant, leaf morphology can show substantial developmental plasticity, which may be evident in response to environmental factors and also as the plant progresses through its life cycle, in a phenomenon called heteroblasty (Poethig, 2010; Zotz *et al*., 2011; Nakayama *et al*., 2017).

One fundamental difference in leaf form is the distinction between simple and compound leaves. In simple leaves, expansion along the primordium margin leads to a single leaf blade, which may be lobed, serrate or entire. Compound leaves consist of a central rachis bearing multiple leaflets, which may be arranged in two basic patterns: as opposite pairs along the rachis (pinnate) or in a terminal fan‐shaped cluster (palmate). In some species, compound leaves exhibit higher orders of complexity or feature substitution of leaflets by other derived organs, such as tendrils. At the cellular level, compound leaf formation involves an extended indeterminacy of the distal tip of the leaf primordium and localized re‐initiation of leaf morphogenic activity along the primordium margin (Efroni *et al*., 2010).

Broadly speaking, one major determinant of leaf shape is the regulation of Class I *KNOTTED1‐LIKE HOMEOBOX* (*KNOX1*) genes. These genes play a crucial role in SAM formation and maintenance across vascular plants (Tsuda & Hake, 2015), and in *Arabidopsis thaliana* and other simple‐leafed species, their expression is stably repressed in developing leaf primordia by orthologs of the *Arabidopsis* MYB domain transcription factor *ASYMMETRIC LEAVES1* (Waites *et al*., 1998; Timmermans *et al*., 1999; Byrne *et al*., 2000; Guo *et al*., 2008). Within the same gene family, Class II *KNOX* (*KNOX2*) genes antagonize *KNOX1* activity by promoting differentiation, and their loss‐of‐function leads to increased leaf complexity similar to *KNOX1* overexpression (Chuck *et al*., 1996; Scofield *et al*., 2008; Furumizu *et al*., 2015). In compound‐leafed species, such as *Solanum lycopersicum* (tomato) and *Cardamine hirsuta* (bittercress), *KNOX1* genes are only transiently repressed and become reactivated in marginal domains to promote lateral leaflet formation (Hay & Tsiantis, 2006; Shani *et al*., 2009). To date, evidence for KNOX2 function in compound leaf species includes gain‐of‐function studies in *Cardamine hirsuta* as well as more recent loss‐of‐function analyses in Medicago and soybean, where *KNOX2* genes were shown to regulate compound leaf patterning and leaflet number (Furumizu *et al*., 2015; X. Wang *et al*., 2023; Clark *et al*., 2026).

Compound‐leafed legume species such as pea (*Pisum sativum*) and *Medicago truncatula* present a distinct scenario, as *KNOX1* genes are not apparently reactivated in leaf primordia (Champagne *et al*., 2007; Zhou *et al*., 2014), and a triple mutant for class I *KNOX* genes does not affect compound leaf morphology (Lu *et al*., 2024). These results suggest that reactivation of meristematic potential necessary for compound leaf development in these species depends on genes other than *KNOX1*. Instead, orthologs of the unrelated transcription factor *LEAFY* (*LFY*) and its co‐factor *UNUSUAL FLORAL ORGANS* (*UFO*) are key regulators, as mutants simplify the compound leaf structure, in addition to their effects on inflorescence meristem identity (Hofer *et al*., 1997; Taylor *et al*., 2001; Dong *et al*., 2005; Wang *et al*., 2008). Recent studies in *Medicago* reveal that the bHLH transcription factor *PINNATE‐LIKE PENTAFOLIATA1* (*PINNA1*) acts upstream of *MtLFY* and *MtUFO* in two distinct mechanisms to control *MtLFY* transcription (He *et al*., 2020) and prevent the activation of *MtUFO* by *KNOX1* genes (Lu *et al*., 2024).

Another important influence on leaf morphogenesis is the *miR156*/*SQUAMOSA PROMOTER BINDING PROTEIN‐LIKE* (*SPL*) module. This was first identified in *Arabidopsis*, where a gradual decrease in *miR156* level and increase in *SPL* activity was shown to drive characteristic age‐related changes in leaf size, shape and tissue patterning (Telfer *et al*., 1997; Wu *et al*., 2009; Manuela & Xu, 2020). This mechanism, sometimes referred to as the phase change or aging pathway, is now known to underlie heteroblasty across angiosperms, including woody trees and herbaceous species (Chuck *et al*., 2007; Wang *et al*., 2011; Silva *et al*., 2019). *MiR156/SPL* regulation also mediates the effects of other age‐related physiological and developmental changes, including organ size, plant architecture, sugar availability and reproductive status (Y. Wang *et al*., 2023; Hussain *et al*., 2024).

In many species, leaf complexity is another feature that increases with developmental age (Ashby, 1948; Tichá, 1985), and the *miR156/SPL* module has also been implicated in this increase (Poethig & Fouracre, 2024). For example, in *Cardamine*, the timing of the developmental transition from simple to compound leaves can be altered by manipulation of miR156/SPL. The interaction between TEOSINTE BRANCHED1/CYCLOIDEA/PROLIFERATING CELL FACTOR (TCP) and CUP‐SHAPED COTYLEDON (CUC) proteins promotes the simple leaf form, but is disrupted by SPL proteins, which release *CUC* transcription factors to promote *KNOX1* expression (Rubio‐Somoza *et al*., 2014). In *Arabidopsis*, *miR156/SPL* may operate through a CUC/TCP complex to switch leaf primordia between KNOX1‐dependent indeterminacy/complexity and KNOX2‐dependent determinacy/simplicity (Challa *et al*., 2021). In *Medicago*, mutants for the *miR156*‐targeted *SPL4* gene exhibit an increase in leaflet number (Min *et al*., 2022), but otherwise little is known about *miR156/SPL* influence in legume compound leaf development.

Pea leaves typically consist of a proximal series of paired leaflets and a distal region in which leaflets are substituted by filiform tendrils, and a well‐known series of mutants has defined key genes specifying compoundness and lateral organ identity (Hofer & Ellis, 1998; Hofer *et al*., 2009; Tayeh *et al*., 2024). Independently of these genes, the number of lateral organ pairs increases with ontogeny (Gould *et al*., 1986). This gradient is influenced by flowering time, and it has been proposed as an expression of heteroblasty (Wiltshire *et al*., 1994), but there is no evidence linking it to the miR156/SPL module (Vander Schoor *et al*., 2022).

To explore the relationship between leaflet number, heteroblasty and flowering time in pea, we searched for determinants of leaflet number using both induced and natural variation and identified several novel loci. We characterize two of these, *EXTRA PINNA PAIRS 1* (*EPP1*) and *EPP2*, describe their genetic interactions with other loci previously shown to alter lateral organ number (*AERO1* and *AERO2*; Taylor & Murfet, 2003; Murfet & Taylor, 2004), and identify putative causal genes for all four. Surprisingly, none of these genes have a clear connection to known genetic modules influencing compound leaf development.

## Materials and Methods

### Plant material and growth conditions

Details and origins of pea (*Pisum sativum* L.) parental lines used in mapping crosses and quantitative trait locus (QTL) populations, and mutant lines used in this study, are listed in Supporting Information Table S1 cv. Térèse (TER) was used for all mapping crosses due to its success in the mapping of several mutants in the TOR/NGB5839 background (Hecht *et al*., 2007, 2011; Liew *et al*., 2014; Sussmilch *et al*., 2015; Ridge *et al*., 2016). TER is sufficiently similar to NGB5839 to minimize segregation of unrelated phenotypic traits while remaining genetically distinct enough to provide adequate marker polymorphism for mapping. All plants were grown in a 1 : 1 mixture of dolerite chips and vermiculite topped with potting mix and received nutrient solution weekly. They were grown in the temperature‐controlled glasshouse in Hobart, Tasmania, under an 18‐h photoperiod consisting of natural daylight extended before dawn and after dusk with *c*. 50 μmol s^−1^ m^−2^ light from sodium vapor lamps. Descriptions of the traits measured in this study are provided in Table S2. EMS‐induced mutants were backcrossed twice to the wild‐type (WT; NGB5839) to reduce background mutations before use in experiments and segregating populations.

### Genotyping and linkage map construction

The three QTL populations (Fig. S1) were genotyped by Diversity Array Technology Pty. Ltd. (Canberra, Australia) using DArTseq markers (Sansaloni *et al*., 2011). Markers were assigned to four quality classes based on call rate, reproducibility, segregation distortion (PIC; Polymorphism Information Content) and average read depth, to assist map curation. For the 5839 × 1794 RIL population, the previously published linkage map was used (Williams *et al*., 2022), with Chromosomes (chr) 1, 4 and 6 inverted to maximize synteny with the *Pisum sativum* L. genome assembly v1a (Kreplak *et al*., 2019). For the remaining populations, linkage maps were constructed using JoinMap v.4 (van Ooijen, 2006).

### QTL analysis

QTL for flowering and leaf change traits were detected using MapQTL v.6 (van Ooijen, 2009). QTL were first identified using *interval mapping* (*IM*), followed by *automatic cofactor selection* (*ACS*). Iterative searches for additional QTL were performed using the *multiple QTL model* (MQM) function, which increases the power of QTL analysis by reducing any residual variance from previously identified QTL (cofactors). The amount of variation explained by each QTL was estimated using the *coefficient of determination* (*R* ^2^) which is represented as the *phenotypic variance explained* (PVE). Appropriate significance thresholds for QTL detection were determined by permutation tests at genome‐wide and chromosome‐wide levels (1000 permutations; Churchill & Doerge, 1994). Putative QTL were defined at two different levels; suggestive (chromosome‐wide type I error rate < 0.05); and significant (genome‐wide type I error rate < 0.05). QTL are represented as a box (1‐LOD interval) and line (2‐LOD interval).

### Fine mapping and marker development

Genomic DNA was extracted from young leaflets using the CTAB extraction protocol (Doyle & Doyle, 1990). For mapping of *AERO1*, *AERO2*, *EPP1 and EPP2* loci, new molecular markers were designed. At the beginning of this research, the pea genome sequence was not available, so markers across this region were designed using *Medicago* synteny, the pea/*Medicago* comparative map (Tayeh *et al*., 2015) and the pea transcriptome (Duarte *et al*., 2014). After 2019, markers were designed using the pea genome (Kreplak *et al*., 2019; Yang *et al*., 2022). Target regions were amplified from genomic DNA by PCR and the products were sequenced using conventional Sanger sequencing. Primers used for PCR and mapping are listed in Tables S3–S6 and were designed from pea sequence using Primer3 (Untergasser *et al*., 2012). For cleaved amplified polymorphic sequence (CAPS) markers, the relevant restriction enzyme is indicated. HRM markers were designed around single‐nucleotide polymorphisms (SNPs) identified between the parental lines. Markers previously developed by other studies are referenced. Sequence analysis and alignment were performed using Geneious Prime (Kearse *et al*., 2012).

Classical and molecular markers were scored in individual plants from the F_2_ generation of the mapping populations (Tables S3–S6). Segregation ratios were tested for deviation from Mendelian expectations. Linkage maps were constructed from estimates of genetic distance between molecular and morphological markers based on segregation data using JoinMap 4 (van Ooijen, 2006).

### Candidate gene identification and RNA‐seq analysis

Potential candidate genes were investigated for each locus by searching regions of interest using the *Medicago* genome (Young *et al*., 2011), the pea transcriptome (Alves‐Carvalho *et al*., 2015), the Cameor genome (Kreplak *et al*., 2019) and the ZW6 genome (Yang *et al*., 2022). We used RNA‐seq of *aero2* and *epp1* to generate enough sequence coverage to look for nonsynonymous SNPs in coding sequence within the fine‐mapped region for each locus. Tissue was pooled from young developing leaves, older fully expanded leaves and shoot apices (*c*. 2 mm) as the gene of interest was expected to be expressed in these tissues. RNA‐seq libraries were sequenced on an Illumina platform to generate paired‐end reads. Reads from mutant and WT samples were mapped independently to the ZW6 genome using Geneious Prime. SNPs were filtered for mutant‐specific variants and C:G>T:A SNPs, which are the mutations most commonly generated by EMS mutagenesis. Putative SNPs were confirmed by checking the aligned reads against the specific gene. A final list of SNPs for both *aero2* and *epp1* is detailed in Table S7. Putative candidate genes were also sequenced using Sanger sequencing of amplified PCR product to check the validity of putative SNPs.

### RNA extraction and quantitative reverse transcription PCR (qRT‐PCR) analysis

Total RNA was extracted from shoot apices collected from 2‐wk‐old pea plants (c. 2 mm wide and 3 mm long) using the SV Total RNA Isolation System (Promega, Madison, WI, USA) according to the manufacturer's instructions. RNA concentration and purity were assessed using a NanoDrop 8000 spectrophotometer (Thermo Fisher Scientific, Waltham, MA, USA). First‐strand cDNA was synthesized from 1 μg of total RNA using the Tetro RT cDNA Synthesis Kit (Meridian Bioscience, Cincinnati, OH, USA). RT‐negative (no reverse transcriptase) controls were included to monitor genomic DNA contamination. Quantitative reverse transcription polymerase chain reaction (qRT‐PCR) was performed using 2 μl of cDNA template and SensiMix SYBR No‐ROX chemistry (Meridian Bioscience, Cincinnati, OH, USA). Reactions were prepared using a Myra robotic liquid handling system (Bio Molecular Systems, Upper Coomera, QLD, Australia) and run on a Rotor‐Gene Q instrument (Qiagen, Hilden, Germany). Cycling conditions consisted of 50 cycles of 95°C for 5 s and 60°C for 20 s. A melt curve analysis was performed to confirm amplification specificity. Transcript abundance was measured using four to six biological replicates and two technical replicates. Relative expression levels were calculated using the ΔΔCt method, normalized to the geometric mean of the reference genes *ACTIN* and *TFIIa* and expressed relative to the WT control. Primer sequences are listed in Table S8.

### Protein sequence, alignment and phylogenetic analysis

For phylogenetic analyses, amino acid sequences for the various species were obtained using Phytozome v.13 (Goodstein *et al*., 2012) or Pulse Crop Database (Humann *et al*., 2019), outlined in Tables S9–S11. Sequences were aligned using MUSCLE v.5 (Edgar, 2022) in Geneious Prime and the phylogenetic trees were obtained using PhyML v.3.3 (Guindon *et al*., 2010) with the LG algorithm and 500 bootstrap branch support.

### Sectioning pea shoot apices

To characterize differences in SAM morphology of the four mutant loci, shoot apices were excised from 2‐wk‐old pea seedlings and fixed under vacuum in 2.5% glutaraldehyde in 0.1 M phosphate buffer, pH 7.2, for 12 h at 4°C. The samples were then dehydrated in an ascending acetone series in 20% increments and taken through three changes of 100% acetone. The apices were slowly infiltrated with Spurr's resin (Spurr, 1969) and polymerizaed blocks were hand‐trimmed with a razor blade. Sections of 4–5 μm thickness were cut with an ultramicrotome. The sections were expanded on a drop of distilled water on a clean glass microscope slide on a moderate hotplate and gently heat‐fixed to the glass. The slides were then immersed in 0.1% (w/v) Toluidine Blue O in 1% (w/v) sodium borate solution for 30s, rinsed in distilled water, decolorized in 70% ethanol for 30s, rinsed again in distilled water and air dried. The sections were mounted in Euparal beneath a coverslip and cured on a cool to moderate hotplate.

## Results

### Pea compound leaves increase in complexity across development

**Fig. 1:**
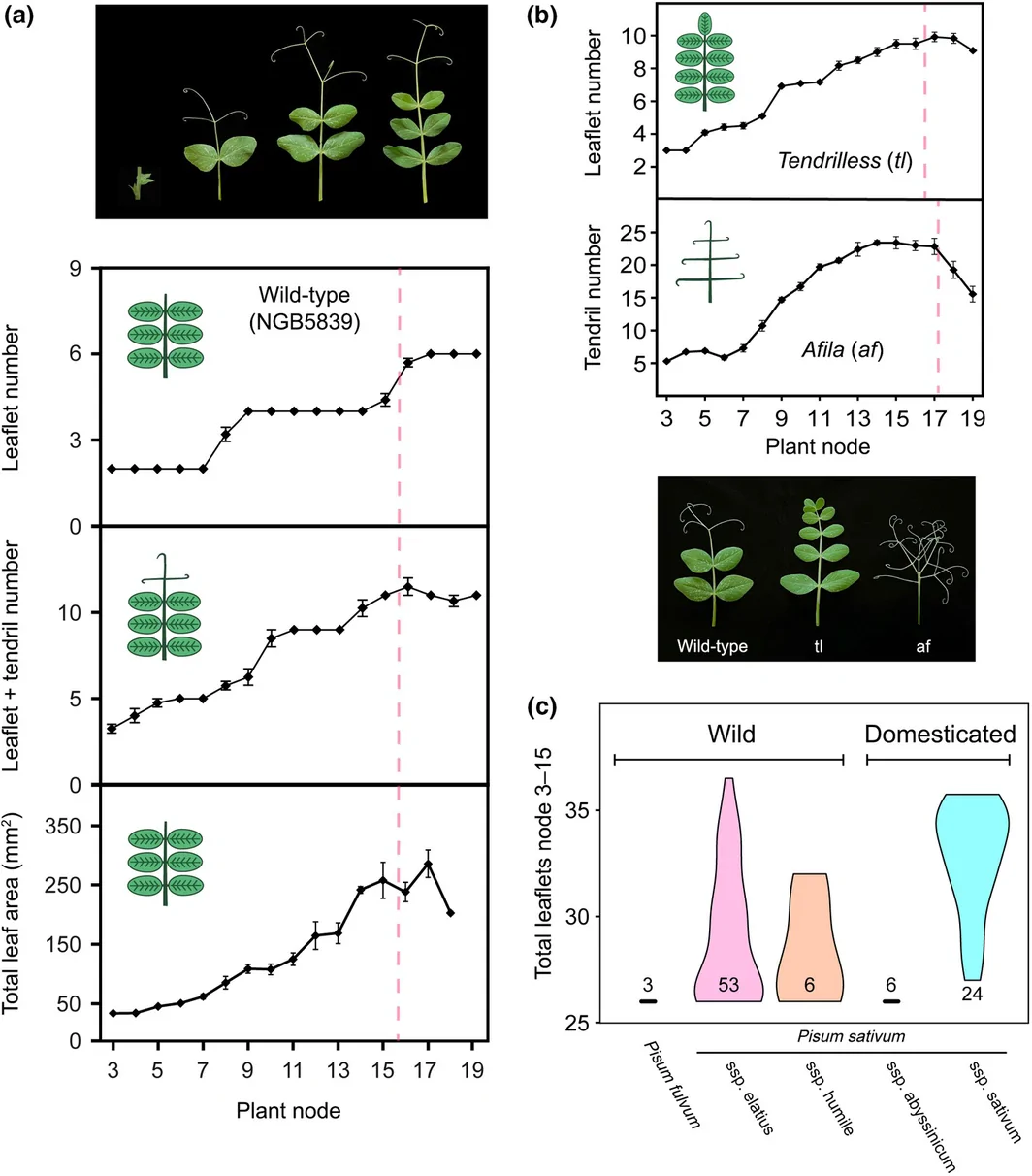
Compound leaf development in pea (*Pisum sativum*). (a) The morphological changes in compound leaf development with age in pea grown under long‐day conditions. The photograph represents the major stages of compound leaf development in pea, from scale leaves through to 6 leaflet +5 tendrils compound leaves. The three panels show the progression of each trait measured in wild‐type (WT) pea (NGB5839; Supporting Information Table S1): Top panel, number of leaflets; middle panel, leaflets and tendrils; and bottom panel, total leaf area. (b) Organ number progression with age in the *tendrilless* (*tl*) and *afila* (*af*) mutants. Representative compound leaves from WT, *tl* and *af* are shown in the photograph. The dotted pink line represents the mean node of flowering for each genotype. Error bars represent the mean ± 1 SE (*n* = 8). (c) A violin plot showing the total number of leaflets from Nodes 3 to 15 in a pea panel containing wild and domesticated pea lines covering the five major groupings in the pea germplasm, *fulvum*, *elatius*, *humile*, *abyssinicum* and *sativum*. Number of individuals per group is indicated on the plot.

Leaves on a typical WT pea plant show progressive changes in morphology across successive nodes (Wiltshire *et al*., 1994), as illustrated in Fig. 1a. The first two nodes produce only small, simple ‘scale’ leaves, but subsequent nodes produce true leaves, which are compound and bear a combination of proximal leaflets and distal tendrils. The first true leaf (at Node 3) bears a single leaflet pair and a rudimentary terminal tendril. Later‐formed leaves develop additional lateral leaflet and tendril pairs, with the transition between even‐leaflet states occurring abruptly (from one node to the next), and leaves with an odd‐leaflet number occurring only occasionally, in a narrow transition zone between even‐leaflet states. Leaves generally reach a maximum of six leaflets (three pairs) and five to seven primary tendrils (two to three pairs plus a terminal tendril) at around the node of flowering although reversion to two leaflet pairs can occur at later reproductive nodes. The number of lateral organs on a leaf will be referred to for convenience as *leaf complexity*, and its change over development of the plant will be referred to as *leaf change*.

Considering both leaflets and tendrils, the total number of primary lateral organs in a leaf also increases but in a more gradual rather than stepwise manner (Fig. 1a middle) and parallels a gradual increase in total leaflet area per leaf (Fig. 1a bottom). This gradual progression in complexity is also evident in mutants in which tendrils are replaced by leaflets (*tendrilless*; Hofer *et al*., 2009) or leaflets by rachides terminating in tendrils (afila; Tayeh *et al*., 2024; Fig. 1b).

A survey of leaf complexity across 95 *Pisum* accessions representing all the major wild and domesticated *P. sativum* subclades showed that in all representatives of the major domesticated subgroup (*P. sativum ssp. sativum*), leaf complexity increased from one to two or three leaflet pairs by Node 15 (Fig. 1c). This is represented by total leaflet number from Nodes 3 to 15, showing that any increase in total leaflet number above 26 leaflets indicates those lines that have transitioned from one leaflet pair to two or more leaflet pairs during this developmental period. By contrast, across the same node interval, leaves in the wild sister species *P. fulvum* and the second minor domesticated subgroup *Pisum sativum* ssp *abyssinicum* never carried more than a single leaflet pair (Fig. 1c). The same was true for the majority of the wild *P. sativum* accessions, including the likely progenitor of ssp. *sativum* (ssp. ‘northern’ *humile*; Hellwig *et al*., 2022) and the more diverse wild ssp. *elatius*. This pattern is consistent with greater leaflet complexity being predominant in the major cultivated pea lineage, although the ancestral leaflet number within Pisum remains difficult to infer because of the complex phylogenetic relationships and extensive introgression among wild and cultivated taxa.

### Genetic control of natural variation for leaf change

**Fig. 2:**
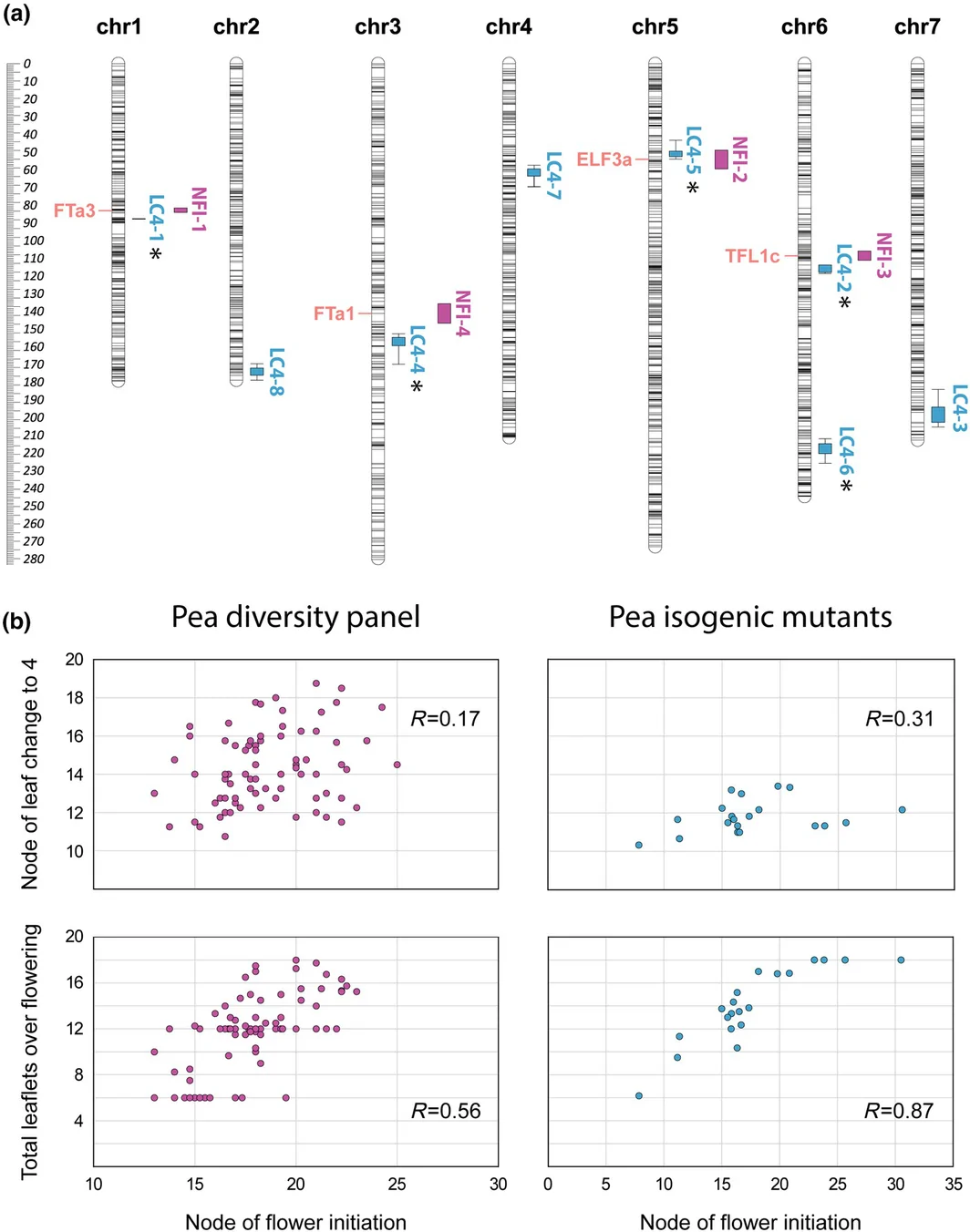
Leaf development correlates with flowering time in pea (*Pisum sativum*). (a) Genetic locations of leaf change and flowering quantitative trait locus (QTL) in the RIL population (NGB5839 × JI1794; Supporting Information Table S1). The positions of four known flowering‐time genes are indicated (Williams *et al*., 2022; *FTa3*, *FTa1*, *ELF3a* and *TFL1c*) QTL for node of leaf change from two to four leaflets (LC4) and node of flower initiation (NFI) are shown on the right of each chromosome and numbered according to the size of the effect or percentage of variation explained (PVE). Asterisks (*) indicate QTL that collocate with leaf change QTL from the other two populations (Fig. S1). (b) Correlation between NFI and two leaf‐development traits: LC4 (top panels) and TL_NFI (total leaflets for three nodes around the node of flowering; bottom panels). Data are shown for a pea diversity panel (left) and pea isogenic mutants (right). Pearson correlation coefficients (R) are indicated on each plot.

The only evidence for genetic control of natural variation for leaflet number in pea has come from a cross between *P*. *sativum ssp. sativum* and *ssp. abyssinicum*, which identified a single major locus on chr3 (Ellis *et al*., 2023). To shed further light on the genetic basis for variation within ssp. *sativum*, QTL analysis for leaflet number was conducted in a wild (ssp. *humile*) × domesticated (ssp. *sativum*) RIL (Recombinant Inbred Line) population (Williams *et al*., 2022). This analysis used the node of transition to four leaflets (LC4) as a simple proxy measurement. This transition occurred 5.4 nodes later in the wild than in the domesticated parent (Table S12), and analysis of the segregating population detected eight QTL, explaining 73.2% of the variation (Fig. 2a; Table S13). A major QTL conferring 48.5% of the variation was located on chr1 (LC4‐1), and the domesticated allele was associated with a promotion of LC4 by 3.8 nodes. The domesticated allele at a second QTL on chr6 (LC4‐2; 7.6% PVE) delayed LC4 by 1.5 nodes. The remaining six QTL were minor, with effects representing 1.6–4.2% PVE.

We next examined two additional F_2_ populations; one in which the same ssp *humile* parent line (JI1794) was crossed to a different domesticated parent (JI1040) and the other a cross between two domesticated lines (NGB5839 × JI2631; Tables S1, S12). Across all three populations we also recorded an additional trait (total leaflet number from Node 3 to 15; TL_3–15). QTL detected in all three populations were represented in a single genomic map based on the physical position of their defining markers (Fig. S1; Tables S14, S15). Three QTL for LC4 were detected in each of the two additional populations. QTL from the second wild × domesticated cross (JI1794 × JI1040) explained 58.8% of the variance, and collocated with loci detected in the RIL cross, including the major locus on chr1 (over 50% of the PVE). By contrast, QTL detected in the domesticated cross (NGB5839 × JI2631) were distinct from those detected in the wild ssp. *humile* × domesticated crosses and were all relatively similar in size with a combined total of 43.8% PVE.

In both wild × domesticated populations, QTL for second leaf change trait (TL_3–15) broadly collocated with QTL for LC4 (Fig. S1; Table S14), identifying the same major locus on chr1 and two other minor QTL. However, in the domesticated cross only a single QTL for TL_3–15 was detected. This did not collocate with any of the three LC4 QTL in this population but did collocate with the major QTL on chr1 from the wild crosses although somewhat weaker (14.7% PVE).

In summary, across all three populations a minimum of 11 distinct genetic regions were associated with leaf change represented by LC4 or TL_3–15. Results from the wild × domesticated populations indicate a single major locus underlying the shift to a greater number of leaflets during domestication, with several smaller‐effect loci also contributing.

### Genetic relationships between leaf change and flowering time

While the molecular and physiological basis for leaf change in pea is unclear, a potential link with the flowering transition has been noted previously (Barber, 1959; Wiltshire *et al*., 1994). Consistent with these previous observations, we found that four of the eight LC4 QTL detected in the initial RIL population (on chr1, chr3, chr5 and chr6) broadly collocated with QTL for the node of flower initiation previously reported in this same population (Williams *et al*., 2022; Fig. 2a). Previous evidence implicated specific flowering genes (*FTa3*, *FTa1*, *ELF3a* and *TFL1c*) as candidates for these four flowering QTL (Williams *et al*., 2022). To further explore the potential contribution of these genes to leaf change, we also evaluated the LC4 trait in specific mutant lines for *FTa1* and *TFL1c* and near‐isogenic lines for *FTa3* and *ELF3a* (Fig. S2; Table S1). Both NFI and LC4 were significantly earlier in the line carrying the *fta3* domesticated allele, indicating that this genomic region influences both flowering and leaf change. However, this was not the case for *fta1* (chr3) or *elf* (chr5; Figs 2a, S2b). Although both mutants significantly altered NFI, they had no significant effect on LC4. This suggests that the loci for LC4 are likely not regulated by these specific flowering loci. Finally, the early‐flowering mutant *tfl1c* also showed earlier LC4 (Fig. S2b).

We also quantified total leaflet number for the three nodes surrounding the node of first flower (TL_NFI) in the RIL population. Because this measure is defined relative to the node of first flower initiation, it standardizes leaflet number to a comparable developmental position across genotypes, thereby reducing variation associated with initiation of flowering. Consistent with this expectation, no QTL for this trait was detected over the major QTL for flowering and LC4 on chr1 (NFI‐1/LC4‐1/*FTa3*) and in a near‐isogenic comparison the *fta3* mutant allele had no significant effect on the TL_NFI trait (Figs 2a, S1, S2a; Table S14). However, we did observe a minor QTL for TL_NFI collocating with NFI‐3/LC4‐2/*TFL1c* on chr6. In addition, the *tfl1c* mutant had significantly fewer leaflets around NFI than the WT (Fig. S2b). Overall, only one of the QTL for TL_NFI was associated with a flowering locus in this population and explained 28% of the total PVE, with the remaining 72% associated with four other regions of the genome (Fig. S1; Table S14) potentially reflecting the action of genes regulating leaf change specifically during the reproductive phase.

Finally, we examined correlations between flowering and leaf change traits in both the diversity panel (Fig. 1c) and an expanded series of isogenic or near‐isogenic pea mutants (Table S16). The correlation of LC4 with NFI in the diversity panel (Fig. 2b, top left) was significant but weak (*r* = 0.27, *P* = 0.018), and no significant correlation was detected in the mutant panel (Fig. 2b, top right; *r* = 0.31, *P* = 0.19). These results indicate substantial independence of these traits in the two contexts. By contrast, strong positive correlations between TL_NFI and NFI were seen in both populations (Fig. 2b bottom; Table S16). Overall, these correlation and collocation results indicate that natural variation in leaf change may partly reflect genetic differences in flowering time, but also a degree of genetic independence from flowering.

### Analysis of induced mutants identifies new loci that influence leaflet number independent of flowering time

**Fig. 3:**
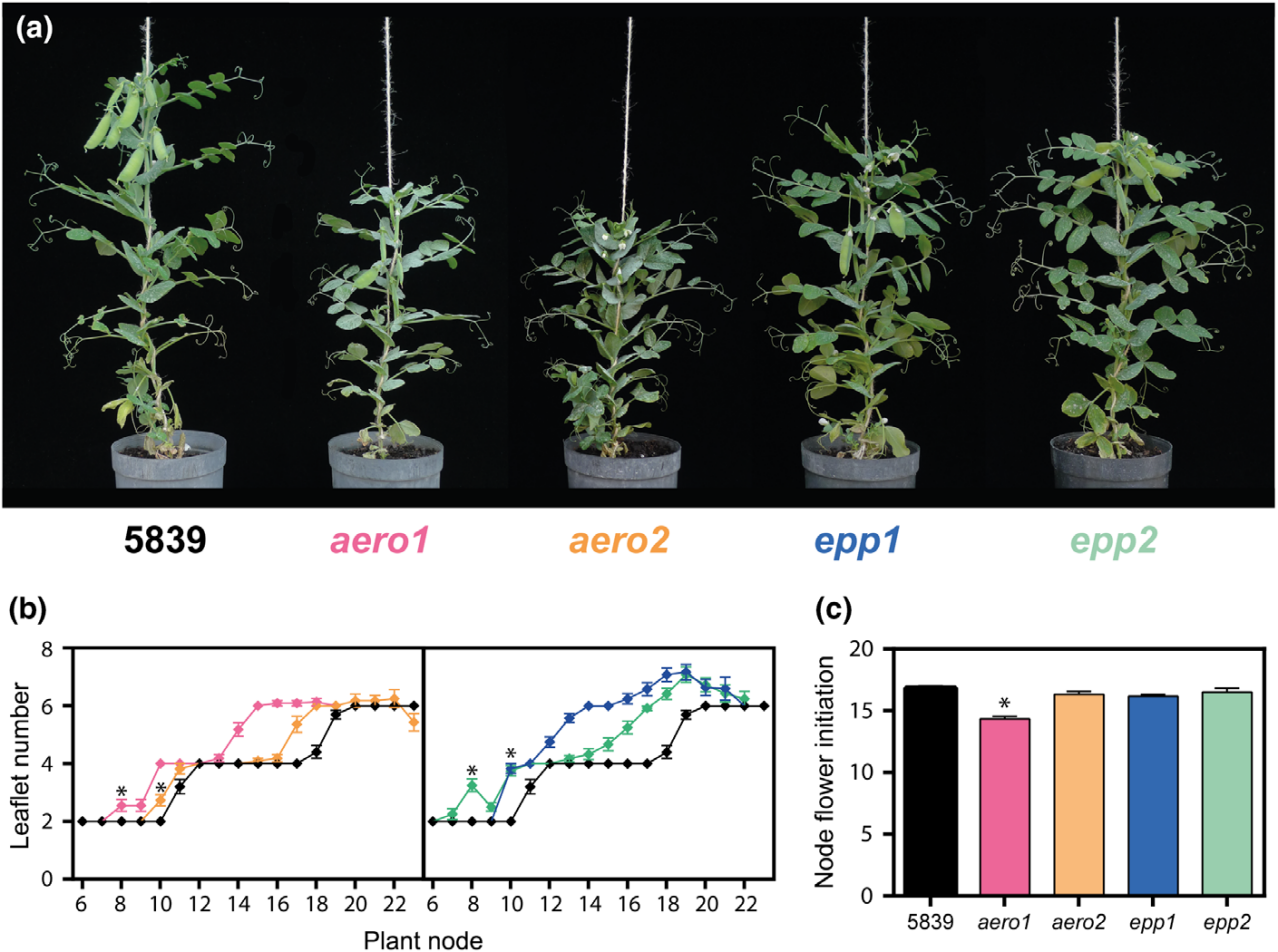
Four mutant loci alter developmental timing in pea (*Pisum sativum*). (a) Wild‐type (WT; NGB5839), *aero1*, *aero2*, *epp1* and *epp2* plants grown under long‐day conditions. Photograph of typical WT and mutant plants taken during the reproductive phase. (b) Total leaflet number scored at each node of plant development, starting at Node 6 until last node is initiated. The WT data for leaflet number are duplicated between panels to allow separate presentation of the two mutant groups. Asterisks (*) denote the first node at which each mutant was significantly different to the WT (*P* < 0.05; Student's *t*‐test). Genotypes are color‐coded based on panel above. (c) Node of flower initiation for the five genotypes, with aero1 significantly different from the other genotypes (*, *P* < 0.01; Student's *t*‐test). Error bars represent the mean ± 1 SE (*n* = 5–6).

In order to identify additional genetic components controlling leaflet number, we screened an EMS‐mutagenized population and isolated two new extra pinna pairs (*EPP*) mutants (Fig. 3; Table S1) in which the change from two to four leaflets, and subsequently to six, occurs at a lower node than WT. In addition, although WT plants never exceed six leaflets under these conditions, the *epp* mutants consistently reach a maximum of eight leaflets (Fig. 3b). These phenotypes are similar to those described previously for *aeromaculata1* (*aero1*) and *aero2* mutants (Taylor & Murfet, 2003; Murfet & Taylor, 2004), although these mutants only occasionally produce leaves with up to eight leaflets (Fig. 3b).

Another contrast is apparent in the stability of leaflet number. The *aero1* and *aero2* mutants retain the ‘stepwise’ change in leaflet number seen in WT plants, with narrow transition zones of one to two nodes between even‐leaflet states, and only rarely produce an intermediate state with an odd‐leaflet number (Figs 1a, 3b). However, in the *epp* mutants, the stability of the transition between these even‐leaflet states is disrupted, with a much greater frequency of odd‐leaflet states and a more gradual increase in leaflet number. In addition, individual *epp* mutant plants exhibited greater variation in the node at which transitions between successive even‐leaflet states occurred. The mutants also showed occasional reversion from higher to lower leaflet numbers throughout development, which in WT is only seen in the final nodes of development as apical arrest occurs (Fig. 3b). Interestingly, whereas the earlier leaf change in *aero1* mutants is paralleled by an earlier flowering node (Taylor & Murfet, 2003; Fig. 3b,c), we did not see a significant effect of *aero2*, *epp1* or *epp2* mutations on reproductive timing (Fig. 3c).

### Mapping of leaf change loci clarifies their relationship and narrows their location

All four mutant loci were next mapped in crosses to cv. Térèse (TER). This line carries the *afila* (*af*) mutation, which alters leaf structure by replacing leaflets with second order pinnae (Tayeh *et al*., 2024), and its use as mapping parent introduced additional complexity into the phenotyping of these populations. However, as shown in Fig. 1b, in the *afila* background the number of terminal tendrils also increases with development, and this trait was used to reliably distinguish WT and *aero*/*epp* mutant segregants in each mapping population (Fig. S3).

**Fig. 4:**
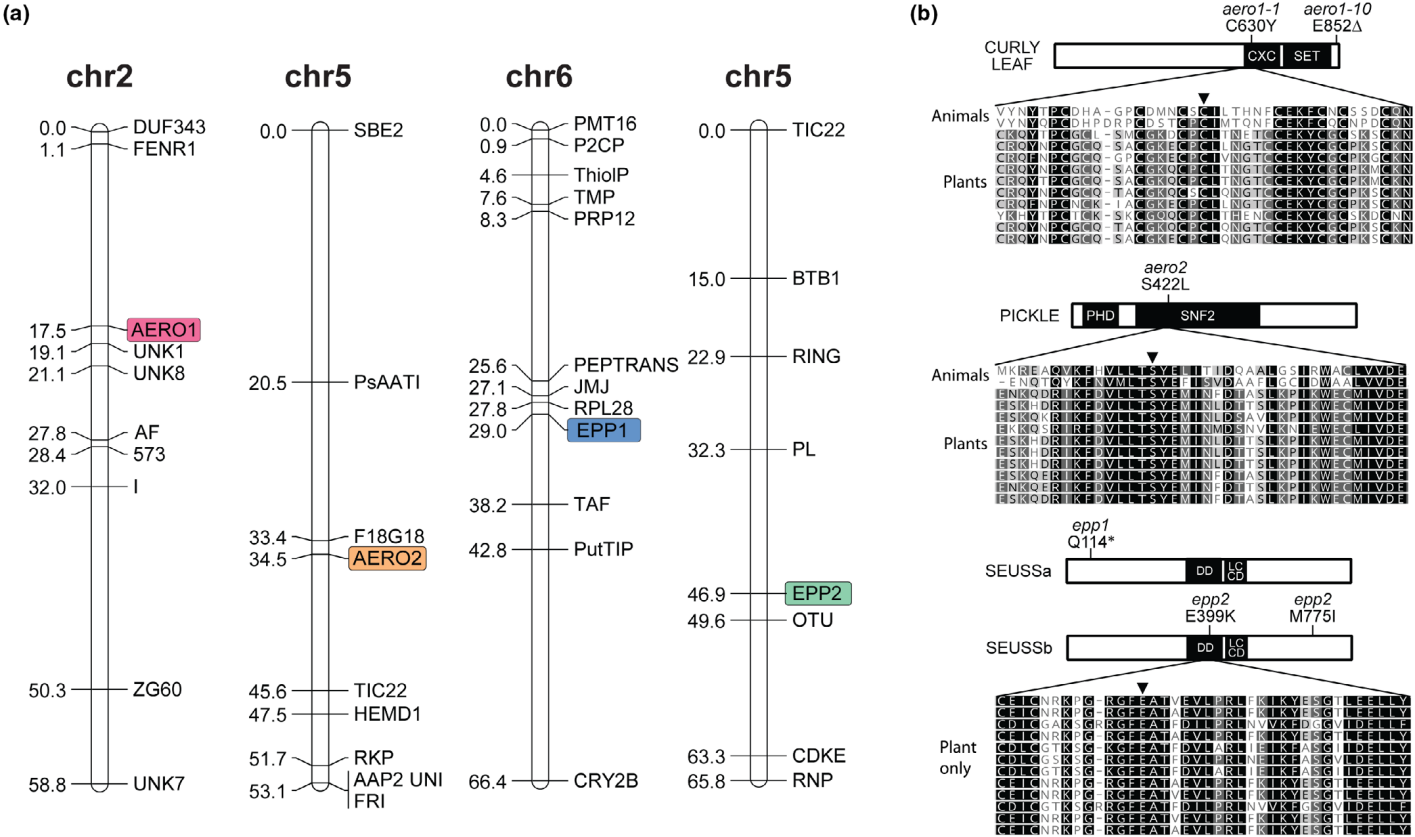
Mapping of the mutant loci and candidate gene identification in pea (*Pisum sativum*). (a) Fine‐mapping results of *aero1*, *aero2*, *epp1* and *epp2* loci using segregating genetic markers in F2 generation of the mutants × cv. Térèse (*n* = 60, 59, 46 and 83 respectively). Details of the markers used in fine mapping are in Supporting Information Tables S3–S6. (b) Putative candidates and the location of each mutation: CURLY LEAF (*aero1‐1* C630Y and *aero1‐10* E852Δ); PICKLE (*aero2* S422L); SEUSSa (*epp1* Q114*); and SEUSSb (*epp2* E399K and M775I). Known functional domains are highlighted with a black box. Domains containing substitution mutations are expanded to show the local protein sequence. A black triangle denotes the amino acid affected.

Early linkage studies initially suggested that *AERO1* was located close to classical markers *AF* and *I* on LGI (chr2; Marx, 1986). We confirmed this position and narrowed the interval to 16.4 cM between markers *FENR1* and *UNK1* (Fig. 4a). Results from the TER × *epp1* F_2_ confirmed an early report of linkage to markers on LG II/chr6 (Refli, 2002) and refined the *EPP1* interval to 10.4 cM between markers *RPL28* and *TAF* (Fig. 4a). A systematic screening of the TER × *aero2* F_2_ (*n* = 83) with published genic markers from Aubert *et al*. (2006) identified linkage with *PsAAT1* on the top of pea LGIII/chr5, and further analysis limited its location to a 12.2 cM interval between markers *F18G18* and *TIC22* (Fig. 4a).

In the case of the *epp2* mutant, we first considered the possibility that it might be an additional allele at *AERO1*, *AERO2* or *EPP1*. However, the *EPP1* marker *JMJ* (Fig. 4a) showed no significant linkage with *epp2* in the TER × *epp2* F_2_ (Fig. S4), clearly establishing that *epp1* and *epp2* represent distinct loci. The *AERO2* flanking marker *TIC22* showed weak linkage with *epp2* (Fig. S4a), suggesting *EPP2* might also be located on chr5, but the large number of recombinants indicated that allelism with *AERO2* was unlikely. Mapping in relation to additional chr5 markers refined the location of *EPP2* to an interval of 17.3 cM between *PL* and *OTU* (Fig. 4a). Overall, these results confirm that *aero1, aero2, epp1* and *epp2* mutants represent four distinct loci.

### Candidate gene analyses identify putative causal mutations for all four loci

We next conducted a thorough search of each genomic interval for candidate genes (Table S7), identifying a number of potential candidates that were evaluated for sequence differences in the mutants. Within the interval was a single‐copy ortholog of *Arabidopsis CURLY LEAF* (*CLF*; Fig. S5). Sequencing of *CLF* cDNA in WT and *aero1‐1* revealed a G1889T transition directing a C630Y substitution in the highly conserved cysteine‐rich domain (CXC; Andersen *et al*., 2007; Fig. 4b). The substitution affects one of the nine cysteine ligands in the first zinc‐binding cluster and is predicted to impair zinc coordination and destabilize the domain structure (Namuswe & Berg, 2012). A second weaker allele, *aero1‐10* (Taylor & Murfet, 2003), carried a single base deletion (T2555∆) resulting in a frameshift 16aa from the C‐terminus, and predicting a further 34 missense aa before termination.

For *AERO2* and *EPP1*, we adopted an RNA‐seq approach, and documented SNPs in candidates for both loci (Table S7). In the case of *AERO2*, only one SNP met the criteria for functional significance, directing a substitution of a highly conserved residue (C1265T, S422L) in a co‐ortholog of the *Arabidopsis* CHD3 chromatin remodeling factor gene *PICKLE* (PKL; Ogas *et al*., 1999; Fig. S6). This residue was perfectly conserved within plant and animal homologs and located within the key Snf2 functional domain (Dürr & Hopfner, 2006; Fig. 4b). For *EPP1*, 167 potentially functionally significant SNPs were identified but only nine were located in plausible candidate genes (Table S7). The most compelling of these was a nonsense mutation (C340T, Q114*) in the first exon of a co‐ortholog of *SEUSS* (*SEU*), an *Arabidopsis* transcriptional co‐regulator (Bao *et al*., 2010; Fig. S7), predicted to eliminate the key dimerization domain (DD) and Ldb1/Chip conserved domain (LCCD) that mediate protein–protein interactions (van Meyel *et al*., 2003; Fig. 4b).

Finally, eight plausible functional candidates were identified in the *EPP2* region, of which one was a second co‐ortholog of *SEUSS* (*SEUSSb*; Fig. S7). This gene carried two mutations in the *epp2* mutant; one (G2872A, E399K) at the end of exon 3 in the DD (Bao *et al*., 2010; Fig. 4b), and a second (G2325A, M775I) in the last exon. Of these two residues, E399 was strongly conserved across plant homologs (Fig. 4b), whereas M775 was not conserved and is represented in *Arabidopsis* SEU by an I (I762), as in the *epp2* mutant. Several EMS‐induced leaf change mutants had previously been identified through phenotypic screening. Following the identification of *epp1* and *epp2* as mutations in SEU homologs, these additional mutant lines were sequenced for candidate mutations. This analysis identified an additional independent line with an *epp* phenotype (Fig. S8a) that also carried a substitution (G1618A; V540I) within the DD of *SEUSSb* and might plausibly represent a second *epp2* allele (*epp2‐2*; Fig. S8b). Consistent with this, *epp2‐2* was clearly more effective in complementation of *epp1* than *epp2* (*epp2‐1*) in their respective F1 combinations (Fig. S8c). A comparison between *epp2‐1* and *epp2‐2* phenotypes suggests that *epp2‐2* might be a weaker allele, although differences in growth conditions between experiments (Figs 3, S8) may have contributed to variation in phenotypic expression. Expression of these candidate genes in excised shoot apices from their respective mutant lines showed no compelling evidence that further implicated them as the likely causal genes, with only *SEUSSa* showing significantly altered expression in *epp1* compared with WT (Fig. S9).

### Expression of compound leaf‐development genes in *aero* and *epp* mutants

To explore the potential molecular consequences of *aero* and *epp* mutations, we also examined their effects on expression analysis of known and potential regulators of compound leaf development in shoot apices from 2‐wk‐old seedlings. These included all five members of the pea class I *KNOX* clade, and the pea *LFY* and *UFO* orthologs (*UNI* and *STP*). Figure S9 shows that there was no consistent pattern of misregulation of these genes across the four mutants, but certain genes showed significant differences in expression. For example, the expression of several *KNOX* genes (*STMa*, *KNAT2* and *KNAT6*) was elevated in the *aero1* mutant, whereas *aero2* showed reduced expression of *STMa* and *KNAT1*. While the functional significance of these differences is not clear, these results overall suggest that the AERO and EPP loci may not share a common molecular mechanism that involves broad transcriptional regulation of any of these known leaf‐development genes, at least at the level of resolution detectable in the tissue we examined.

### Inheritance and interactions between *epp1* and *epp2* mutants

Given that *EPP1* and *EPP2* are likely SEU paralogs, characterizing their inheritance and potential genetic interaction was of interest. Fig. S10a shows that heterozygous WT × *epp1* F_1_ heterozygous plants (*epp1*/+; 66.82 ± 0.5) were more similar to WT (61.86 ± 1.4) than to *epp1* (82.75 ± 1.1) but were still significantly different from WT (*P* < 0.05), indicating incomplete dominance. *epp2*/+ plants (71.75 ± 1) also differed from WT (*P* < 0.05), but in contrast to *epp1*/+, were much more similar to the *epp2* parent (75 ± 1) than to WT, although still with significantly fewer leaflets (*P* < 0.05). This indicates a lower degree of dominance for the WT *EPP2* allele in comparison with *EPP1*, and a tendency toward dominant inheritance of the *epp2* mutation, which is also reflected in the trait distributions for their respective F_2_ populations (Fig. S10b).

Interestingly, total leaflet number in *epp1*/+ *epp2*/+ plants (i.e. doubly heterozygous for *epp1* and *epp2*; Fig. S10a) exceeded either single mutant in range (62–100 vs 79–88 for *epp1* and 72–81 for *epp2*), but the mean was significantly higher than either single heterozygote (82.9 ± 1.4, *P* < 0.05 in both comparisons). In addition, in the F_2_ progeny (data not shown), only three individuals of 66 had a WT phenotype, consistent with the expected proportion of individuals homozygous for WT alleles at both loci. Together, these results show that despite being nonallelic, the *epp1* and *epp2* mutations fail to complement each other. This is consistent with dosage sensitive partial redundancy and suggests that both genes contribute to the same molecular process.

**Fig. 5:**
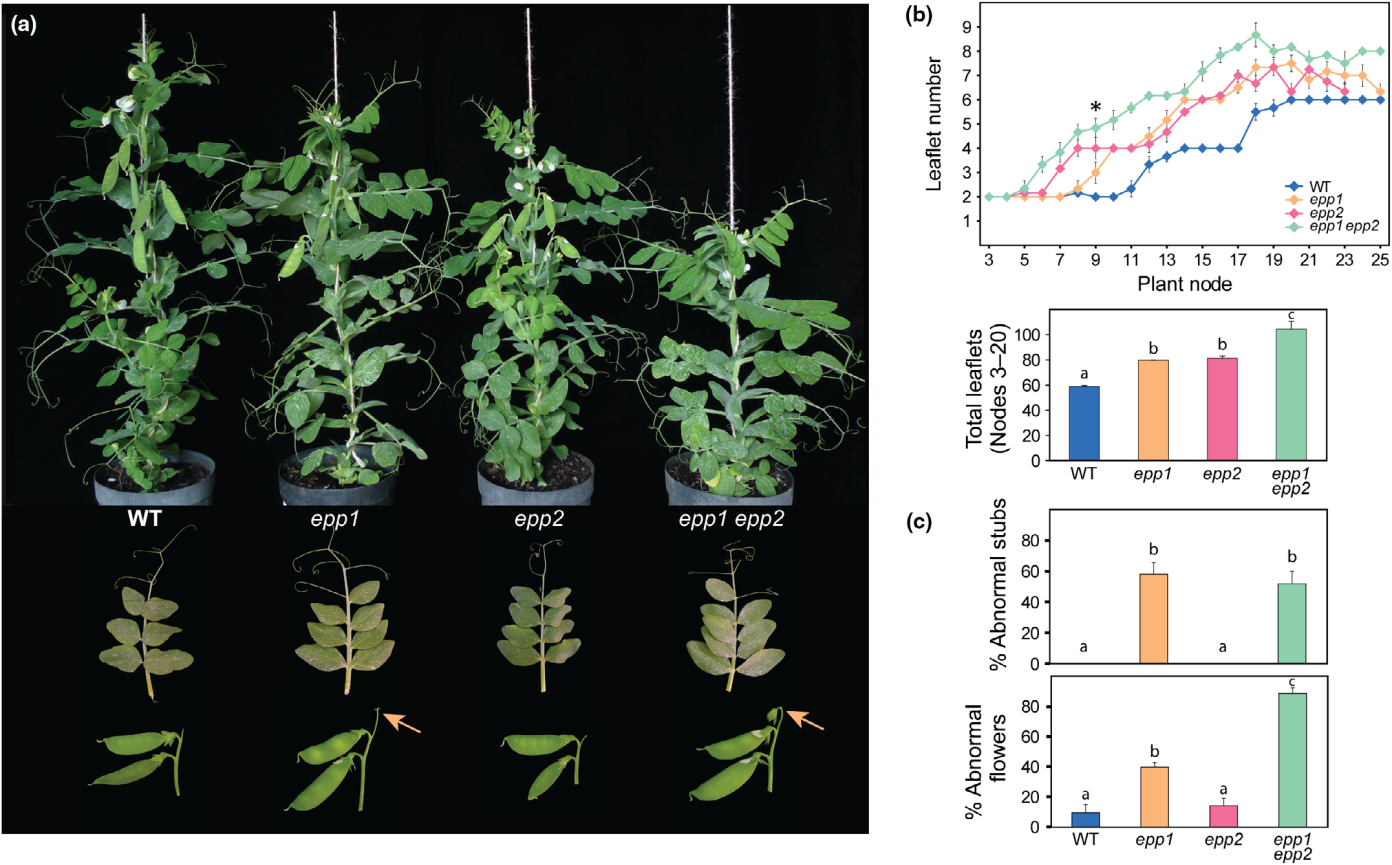
*Epp1* and *epp2* mutant interactions in pea (*Pisum sativum*). (a) Photographs of wild‐type (WT; NGB5839), *epp1*, *epp2* and *epp1 epp2* double mutant plants grown under long‐day conditions during the reproductive phase. Leaves were taken from Node 18 of each genotype. Reproductive structures show the indeterminate stub with additional aborted flowers or vegetative structures as seen in *epp1* mutants (indicated by orange arrows). (b) The number of leaflets at each node starting at Node 3. Asterisk (*) denotes the first node at which all mutants were significantly different to the WT (*P* < 0.05). The average total leaflet number (Nodes 3–20) for each genotype is also shown. (c) Reproductive and floral structure abnormalities are represented by % abnormal stubs and % abnormal flowers, respectively. Error bars represent the mean ± 1 SE (*n* = 6). Tukey's letters denote significant differences (*P* < 0.05; Tukey's HSD).

Molecular genotyping in the F_2_ identified all four homozygous genotypes (Fig. 5). Overall, the *epp1 epp2* double mutant was notably more extreme than either single mutant in terms of total leaflet number and leaflet number at each individual node (Fig. 5b). In addition, the maximum leaflet number in the double mutant often reached 9 or 10 leaflets rather than seven or eight in the single mutants (Fig. 5a,b). Beyond differences in leaflet number, we also consistently observed defects in flower and inflorescence structure. The single *epp1* mutant showed evidence of reduced determinacy in the secondary inflorescence structure (Fig. 5a,c; Sussmilch *et al*., 2015) and incomplete specification of floral organ identity and separation in the outer two whorls (Fig. 5c). Although the *epp2* single mutant showed none of these defects, the *epp2* mutation did enhance the floral abnormalities in the *epp1* mutant (Fig. 5c).

### Double and triple mutant phenotypes display additive and enhanced genetic interactions across multiple areas of plant development

Given the similarity of leaf change phenotypes in *epp* and *aero* mutants, we next explored the interaction of *epp1* with both *aero1* and *aero2*, by selecting the triple mutant and all double mutant combinations from several segregating progenies. Phenotypes of all genotypic combinations are summarized in Table S17.

**Fig. 6:**
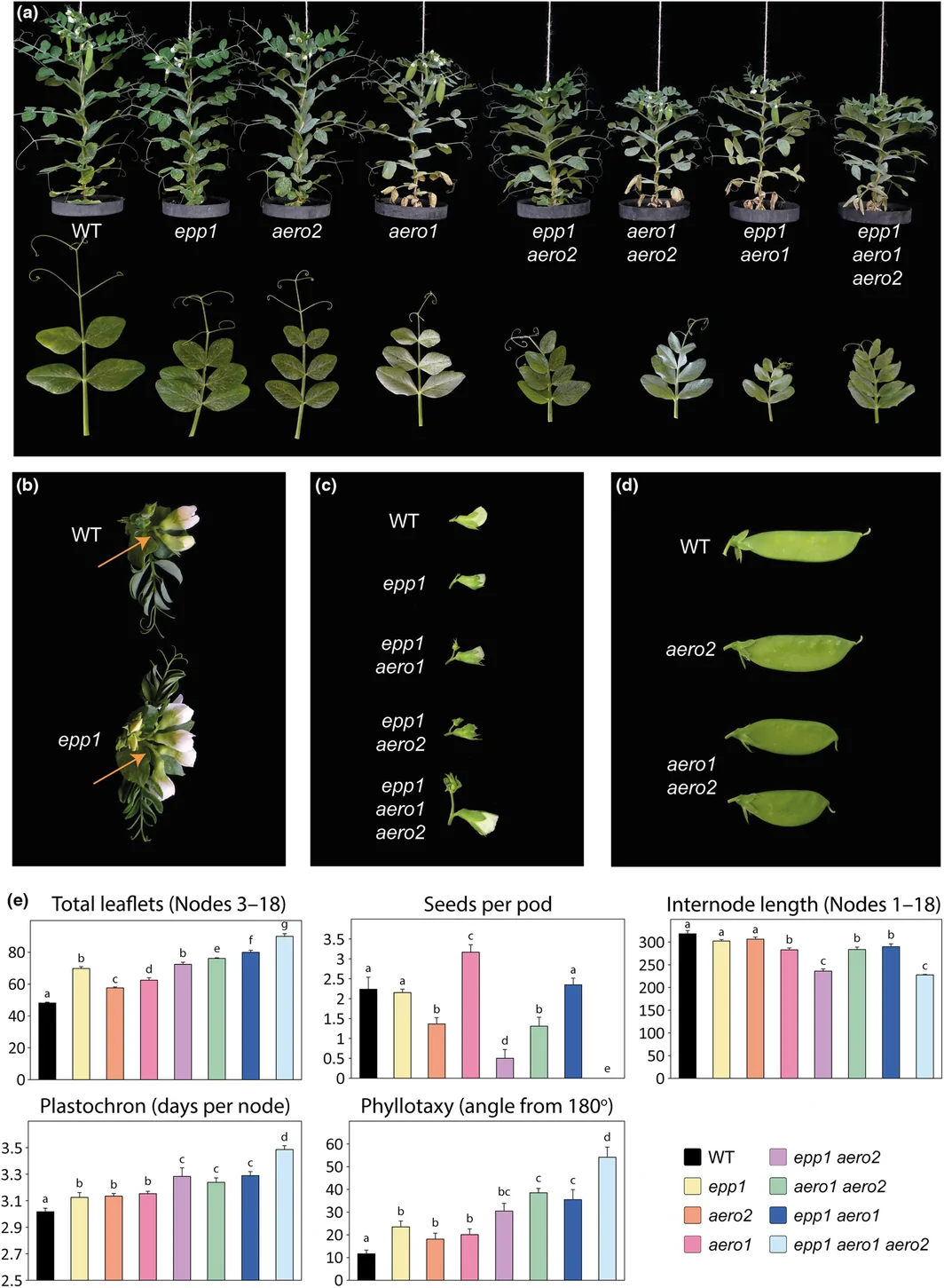
Double and triple mutant phenotypes in pea (*Pisum sativum*) reveal additive and enhanced interactions. (a) Single, double and triple mutant plants photographed at the reproductive stage with a representative leaflet shown for each genotype below from Node 15 showing increased complexity with added mutations. (b) Example of perturbed phyllotaxy in the mutant *epp1*. Orange arrows indicate multiple inflorescences arising from a single node in *epp1* compared with a single inflorescence in wild‐type (WT). (c) Examples of reproductive architecture defects in *epp1* mutants. (d) Examples of pod defects in *aero2* mutants. (e) Measurements of traits that were altered in the single, double and triple mutant combinations: Total leaflets from Nodes 3 to 18; Number of seeds per pod; Internode length from Nodes 1 to 18; Plastochron rate calculated at 6 wk of age; Phyllotaxy measured as relative leaf positioning. In a WT plant, leaves develop sequentially on the opposite side (180°) from the previous leaf. We measured the leaf angle difference from 180° in relation to the previous leaf for the top 10 nodes of plant. Error bars represent the mean ± 1 SE (*n* = 4–8). Tukey's letters denote significant differences (*P* < 0.05; Tukey's HSD).

Two of these traits have previously been reported as characteristic of *aero* mutants; increased leaf flecking in *aero1* and *aero2*, and early flowering in *aero1* (Sidorova & Uzhintseva, 1995; Taylor & Murfet, 2003; Fig. 3c). The effects of *aero1* and *aero2* on flecking were additive but were not substantially enhanced by the addition of the *epp1* mutation (Fig. 6a). Likewise, early flowering of *aero1* was not enhanced by either *aero2* or *epp1*.

For other phenotypes shown in Table S17, defects generally become more severe as these mutations are combined. For example, maximum leaflet number and total leaflet number in *aero1 aero2* and *aero1 epp1* double mutants were significantly higher than in the single mutants and also significantly higher in the triple mutant than in any of the double mutants (Fig. 6a,e). Like the *epp1 epp2* double mutant (Fig. 5), the triple *epp1 aero1 aero2* mutant also had 9 to 10 leaflets (Fig. 6a).

A similar trend is also seen for other traits previously reported in the *aero2* mutant, including pod shape, number of seeds per pod and internode length (Murfet & Taylor, 2004), with mutant combinations in general conferring increasingly shorter and deeper pods with fewer seeds (Fig. 6d,e), and shorter internodes (Fig. 6a,e). The floral defects and reduced inflorescence determinacy in the *epp1* mutant (Fig. 5) were also similarly enhanced by the *aero1* and *aero2* mutations (Fig. 6c).

**Fig. 7:**
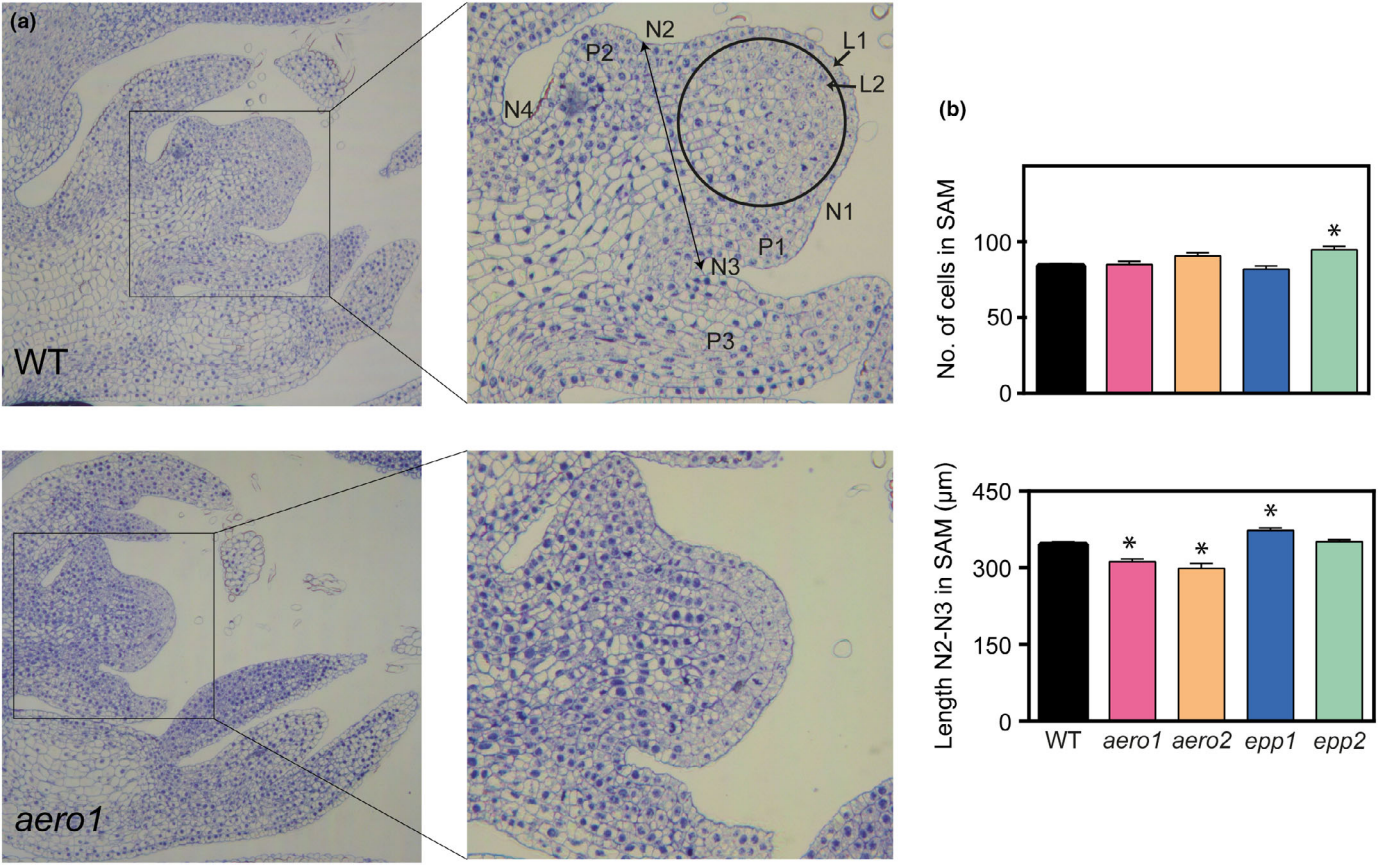
Mutants in pea (*Pisum sativum*) exhibit perturbed apex and leaf primordia development. (a) Shoot apical meristem (SAM) sections of wild‐type (WT) and aero1. Plants were 2 wk old, and the youngest leaf primordia bulge (P1) is Node 14. There was no difference in the rate of leaf development at this young stage between the genotypes. On the left is the 10× image, and the black box indicates the area on the right that is enhanced to 20× to show the cells in apical meristem and the youngest leaf primordia developing. (b) Measurements of cell number and length in the mutants. The number of cells in the apical meristem (AM) was determined using a 200 μm diameter circle with the edge of the circle aligned to the boundary between the L1 and L2 cell layers. The length of the N2–N3 was determined by measuring the distance (μm) between the proximal adaxial point of leaf primordia 2 (P2) to the proximal adaxial point of leaf primordia 3 (P3). Error bars represent the mean ± 1 SE (*n* = 4). Any significant differences from the WT are highlighted with an asterisk (*, *P* < 0.05).

Other developmental abnormalities also became apparent, including a slower rate of development (increased plastochron) and alterations to phyllotaxy. Detailed measurements revealed subtle but significant defects for both traits in all three single mutants, and additive effects as mutations were combined (Fig. 6e). In the *epp1* mutant in particular, the phyllotaxy defects appeared to be associated with an altered apical meristem organization (Fig. 6b,e) typical of mild fasciation (Choob & Sinyushin, 2012). Since these visible defects are likely to reflect changes in shoot apex earlier in development, we examined morphology of the shoot apex in single mutants (Fig. 7) at a relatively early timepoint (2‐wk‐old plants, initiation of leaf 14 primordia) where the leaflet number phenotype is clearly expressed but there is not yet any clear alteration to phyllotaxy. We observed subtle defects that included an apparent flattening in *aero1* and *aero2*, a more strongly domed shape in *epp1*. However, except for *epp2*, the number of cells in the apical meristem of mutants was not significantly different from WT (Fig. 7). While measurements from histological sections should be interpreted with caution, these observations are consistent with the possibility that the mutants all affect basic developmental properties of the shoot apex.

## Discussion

The genetic control of leaf development is well defined in the simple‐leafed model species *Arabidopsis*, and work in species such as *Cardamine* and tomato is yielding growing insight into the mechanisms through which more complex leaf forms are specified (Ali *et al*., 2020; Bhatia *et al*., 2023; Hu *et al*., 2023). Legumes are another major plant group in which compound leaves are widespread, and studies in pea and *Medicago* are revealing distinctive features of their control (Hofer & Ellis, 1998; DeMason & Villani, 2001; Mo *et al*., 2022; He *et al*., 2026). A central aim of this study was to discover new genetic components in the regulation of legume compound leaf morphology. Surprisingly, our provisional molecular identification of the loci suggests that all four are orthologs of genes with broad roles in the control of gene expression. CLF and PKL are epigenetic regulators associated with Polycomb Repressive Complex 2 and H3K27 methylation (He *et al*., 2013; Xiao & Wagner, 2015), while SEU and SEU‐LIKE proteins encode transcriptional co‐regulators that act as adaptors in gene regulatory complexes and are even known to recruit chromatin modifiers in some contexts (Bao *et al*., 2010; Zhai *et al*., 2020). While none of these have been directly associated with age‐related changes in leaf morphology or compound leaf development in other species, the general nature of their inferred functions make it plausible that they might regulate genes known to be acting in these leaf‐development pathways.

Evidence on *CLF*, *PKL* and *SEU* function in *Arabidopsis* provides some clues to potential mechanisms that could underlie their effects in pea. For example, *Arabidopsis CLF* represses genes with roles in peripheral meristems and differentiating leaves, including class I *KNOX* genes, such as *STM*, *KNAT1* and *KNAT2* (Katz *et al*., 2004; Schubert *et al*., 2006; Shen & Xu, 2009). *Arabidopsis SEU* and its co‐repressor *LEUNIG* (*LUG*) also act together to suppress *STM* expression and to sustain meristematic potential in lateral organs (Cnops *et al*., 2004; Stahle *et al*., 2009; Bao *et al*., 2010). Thus, both *CLF* and *SEU* may contribute to the repression in leaves of genes such as *STM* that act to confer meristematic competence. Recent evidence also implicates *PKL* in maintaining stable repression of PRC2 targets (Liang *et al*., 2024). Taken together with the report that ectopic *STM* expression in *Arabidopsis* leaves can (under specific circumstances) cause increased lobing (Aguilar‐Martinez *et al*., 2007), these observations provide a plausible mechanistic link between the *AERO* and *EPP* genes and their observed effects on leaf structure in pea.

However, previous studies in compound‐leaved legumes, primarily in pea and *Medicago truncatula*, have identified a number of additional components in the control of compound leaf development that may substitute for *KNOX* genes as central regulatory targets. *LFY* orthologs *UNI*/*SGL* delay terminal differentiation of leaf primordia, and in loss‐of‐function mutants compound leaf replaced by single leaflet (Hofer *et al*., 1997; Wang *et al*., 2008). Similarly, *STP*/*UFO* orthologs extend indeterminacy, as mutants have fewer lateral organs than WT in developmentally equivalent leaves, and overexpression results in additional pair of lateral leaflets (Taylor *et al*., 2001; Lu *et al*., 2024). Mutations in the *BELL‐like homeobox* (*BLH*) gene *PINNA1* also confer an additional leaflet pair phenotype which is dependent on functional UFO and KNOX I proteins and reflects a KNOX1‐dependent repression of UFO by PINNA1 (Lu *et al*., 2024).

The *aero* and *epp* mutants described here are thus broadly similar to *pinna1* mutants, in the sense that they appear to extend the window for indeterminate growth of the leaf primordium in the proximal‐distal axis, allowing the formation of additional lateral organs on the primary leaf rachis. Similar to *pinna1* mutants, they do not appear to affect complexity or ‘compoundness’ per se, as neither single mutants nor mutant combinations have any effect on the degree of leaf dissection or on leaflet morphology, other than a compensatory reduction in leaflet size. This similarity suggests alternative potential mechanisms for action of *AERO* and *EPP* genes, through repression of *STP*. A role for the *PINNA1* ortholog in pea has yet to be determined, but it is also plausible *AERO* and *EPP* genes might also regulate *STP* indirectly via repression of *KNOX* or promotion of *PINNA1* activity. Conversely, functional analysis of *EPP/SEU* genes in *Medicago* may provide another useful point of comparison between these systems.

While the effects of all four mutations on leaflet number are broadly similar, there is a characteristic difference between *aero* and *epp* mutants in the transition between even‐number leaflet states, which appears less robust in *epp* mutants (Fig. 3). This may point to a difference in their developmental roles. One possibility is that both *AERO* and *EPP* genes may accelerate the addition of leaflet pairs by increasing the indeterminacy of the leaf primordium, but *EPP* genes may have an additional role in specifying the identity or fate of individual leaflet primordia as tendril or leaflet. The significance of this distinction will depend on more detailed future investigations of the molecular mechanisms underlying *AERO* and *EPP* effects.

Changes in leaf size, shape and morphology have also been linked to age‐related changes in expression of the *miR156–SPL–miR172* pathway (Wang *et al*., 2011; Poethig, 2013), and this pathway has also been implicated in leaflet‐related changes in legumes (Min *et al*., 2022). However, our previous work in pea showed that leaf change does not coincide with changes in expression of pathway components (Vander Schoor *et al*., 2022), and the identities of *aero* and *epp* mutants reported here also suggest no specific connection between leaflet number and *miR156–SPL–miR172* regulation. While there is some evidence that this pathway is subject to epigenetic control involving *PKL* and *CLF* in *Arabidopsis* (Xu *et al*., 2018), there is no clear evidence for control by *SEU*, and thus for any obvious or direct mechanistic connection to the aging pathway across the four pea loci.

Nor does there appear to be any consistent relationship between leaf change and flowering time, since in three of the four mutants (*aero2*, *epp1*, *epp2*) flowering time is not affected (Fig. 3c). However, an association between leaflet number and flowering time is detected in our analyses of natural variation, with major‐effect loci for leaf change co‐locating with known flowering‐time loci/genes (Fig. 2a; Williams *et al*., 2022). Additionally, near‐isogenic lines for these flowering‐time loci also displayed altered leaf change implying that the flowering‐time loci may have a direct regulatory influence on leaf complexity (Fig. S2). A similar mechanistic relationship has been shown in other species such as *Cardamine hirsuta*, where natural variation in the flowering‐time repressor *ChFLC* drives both accelerated flowering and accelerated leaf complexity (Cartolano *et al*., 2015). In the *Cardamine* system, this connection reflects an effect of *FLC* on components of the aging pathway and is interpreted as a mechanism for optimizing leaf growth in relation to life cycle (Cartolano *et al*., 2015). It might also be interpreted more generally in terms of pleiotropic effects of florigen genes on resource allocation (Shalit *et al*., 2009).

Beyond this consideration of specific pathways, our observations suggest that changes in leaflet number, although distinctive, may simply represent a threshold effect imposed on an underlying gradient in leaf developmental potential. This gradient is evident both from the more gradual increase in total leaflet area in WT plants and in lateral organ number in ‘leafless’ *af* genotypes (Fig. 1). Under this model, later formed and better‐resourced leaf primordia would have an increased growth potential at the distal end of the primary axis, allowing additional sites for lateral organ initiation. It is therefore conceivable that age‐related changes in leaf form could be determined through the interaction between several developmental and metabolic processes that converge on meristematic activity. These might include sugar and hormone signaling and cell cycle regulation, as well as pathways controlling flowering, aging and leaf development. A better understanding is likely to require a deeper characterization of mutant phenotypes, including potential effects on these pathways and processes, and parallel studies in other compound‐leafed species.

## Competing interests

None declared.

## Author contributions

JLW, JKVS, VH and RJEW planned and designed the research. JKVS, CJB, WG, JLW and RJEW performed the experiments. GA and JB provided sequence data. JKVS, JLW and VH analyzed the data. JKVS and JLW wrote the manuscript.

## Disclaimer

The New Phytologist Foundation remains neutral with regard to jurisdictional claims in maps and in any institutional affiliations.

## Supporting information


**Fig. S1** Genomic locations of QTL for leaf development in pea (*Pisum sativum*).
**Fig. S2** Leaf change traits in pea (*Pisum sativum*) flowering mutants.
**Fig. S3** Identification of *epp1 afila* double mutant phenotype in pea (*Pisum sativum*).
**Fig. S4** *A*llelism testing of *epp2* in pea (*Pisum sativum*).
**Fig. S5** Phylogenetic relationships of CURLY LEAF family proteins.
**Fig. S6** Phylogenetic relationships of PICKLE family proteins.
**Fig. S7** Phylogenetic relationships of SEUSS family proteins.
**Fig. S8** Characterization, molecular analysis and allelism testing of the *epp2‐2* allele in pea (*Pisum sativum*).
**Fig. S9** Expression of candidate and compound leaf‐development genes in *aero* and *epp* mutants in pea (*Pisum sativum*).
**Fig. S10** Pea (*Pisum sativum*) *epp1* and *epp2* inheritance patterns.

**Table S1** Pea (*Pisum sativum*) genotypes used in this study.
**Table S2** Plant phenotyping details.
**Table S3** *AERO1* locus mapping markers.
**Table S4** *AERO2* locus mapping markers.
**Table S5** *EPP1* locus mapping markers.
**Table S6** *EPP2* locus mapping markers.
**Table S7** Candidate genes for *AERO* & *EPP* loci in pea (*Pisum sativum*).
**Table S8** Pea (*Pisum sativum*) primers used in this study.
**Table S9** CURLY LEAF and related E(z) family protein sequences.
**Table S10** PICKLE and PKL‐related protein sequences.
**Table S11** SEUSS and SEU‐like related protein sequences.
**Table S12** Parent leaf change phenotypes.
**Table S13** QTL data for Fig. 2a.
**Table S14** QTL data for Fig. S1.
**Table S15** Candidate gene list for Fig. S1.
**Table S16** Correlation tables of flowering and leaf change traits in pea (*Pisum sativum*).
**Table S17** Summary of trait defects observed in single, double and triple mutant pea (*Pisum sativum*) genotypes.
Please note: Wiley is not responsible for the content or functionality of any Supporting Information supplied by the authors. Any queries (other than missing material) should be directed to the *New Phytologist* Central Office.

## Data Availability

Protein and gene sequences used for alignment and phylogenetic analyses were obtained from publicly available databases. Accession numbers and source databases (NCBI, Phytozome, Pulse Crop Database) are listed in Tables S9–S11. All other data supporting the findings of this study are provided in the Supporting Information: pea genotypes used (Table S1), plant trait details (Table S2), fine‐mapping markers (Tables S3–S6), candidate gene lists (Tables S7, S15), primer sequences (Table S8), protein sequences for phylogenetic analyses (Tables S9–S11), parent phenotypes (Table S12), QTL data (Tables S13, S14), correlation analyses (Table S16) and trait defect summary (Table S17).
