# Supplementary material for: New components in genetic regulation of age‐dependent compound leaf architecture in pea
Source: New Phytol. 2026 Aug 14;252(2):909–25. doi: 10.1111/nph.71515 (PMC13583374; doi:10.1111/nph.71515)
Supplement: Supplementary file 1 [file NPH-252-909-s001.pdf]

New Phytologist

New Phytologist Supporting Information

Article title: **New components in genetic regulation of age-dependent compound leaf architecture in pea.**

Authors: Jacqueline K. Vander Schoor, Chantelle J. Beagley, Warwick Gill, Grégoire Aubert, Judith Burstin, Robert J.E. Wiltshire, Valérie Hecht, James L. Weller

Article acceptance date: 30 July 2026

The following Supporting Information is available for this article:

Fig. S1 Genomic locations of QTL for leaf development in pea (*Pisum sativum*)
Fig. S2 Leaf change traits in pea (*Pisum sativum*) flowering mutants
Fig. S3 Identification of *epp1 afila* double mutant phenotype in pea (*Pisum sativum*)
Fig. S4 *A*llelism testing of *epp2* in pea (*Pisum sativum*)
Fig. S5 Phylogenetic relationships of CURLY LEAF family proteins
Fig. S6 Phylogenetic relationships of PICKLE family proteins
Fig. S7 Phylogenetic relationships of SEUSS family proteins
Fig. S8 Characterisation, molecular analysis and allelism testing of the *epp2-2* allele in pea (*Pisum sativum*)
Fig. S9 Expression of candidate and compound leaf development genes in *aero* and *epp* mutants in pea (*Pisum sativum*)
Fig. S10 Pea (*Pisum sativum*) *epp1* and *epp2* inheritance patterns

Tables in the additional excel file:

Table S1 Pea (*Pisum sativum*) genotypes used in this study
Table S2 Plant phenotyping details
Table S3 *AERO1* locus mapping markers
Table S4 *AERO2* locus mapping markers
Table S5 *EPP1* locus mapping markers
Table S6 *EPP2* locus mapping markers
Table S7 Candidate genes for *AERO* & *EPP* loci in pea (*Pisum sativum*)
Table S8 Pea (*Pisum sativum*) primers used in this study
Table S9 CURLY LEAF and related E(z) family protein sequences
Table S10 PICKLE and PKL-related protein sequences
Table S11 SEUSS and SEU-like related protein sequences
Table S12 Parent leaf change phenotypes
Table S13 QTL data for Fig. 2a
Table S14 QTL data for Fig. S1
Table S15 Candidate gene list for Fig. S1
Table S16 Correlation tables of flowering and leaf change traits in pea (*Pisum sativum*)
Table S17 Summary of trait defects observed in single, double and triple mutant pea (*Pisum sativum*) genotypes

Fig. S1 Genomic locations of QTL for leaf development in pea (*Pisum sativum*)

QTL identified across seven pea chromosomes from three mapping populations: Population 1 (*NGB5839 × JI1794*, F_8 RIL), Population 2 (*NGB5839 × JI2631*, F_2), and Population 3 (*JI1794 × JI1040*, F_2). Bars (1-LOD interval) and lines (2-LOD interval) indicate QTL positions. Traits measured are LC4 (node of leaf change from two to 4 leaflets), TL_3-15 (total leaflets from node 3 to 15) and TL_NFI (total number of leaflets for 3 nodes surrounding the flowering node). The QTL are numbered according to the size of the effect with 1 being the highest PVE (Percentage of variation explained). The genomic position of candidate genes from published studies are indicated on each chromosome and details of the candidate genes are in Table S15. The LC4 QTL are highlighted for each population using a coloured box.

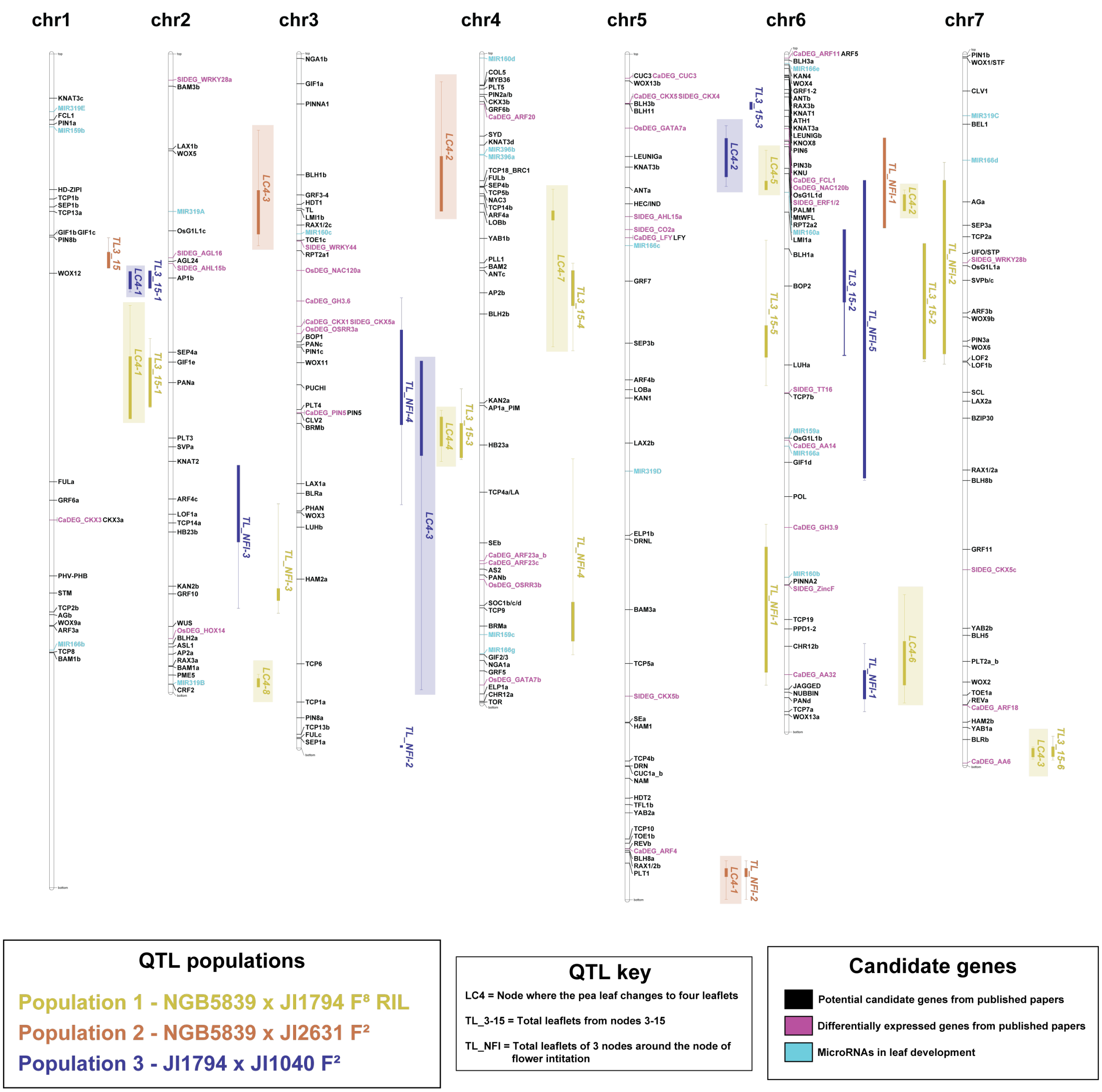

Fig. S2 Leaf change traits in pea (*Pisum sativum*) flowering mutants
(a) Comparison of FTA3 wild-type (WT) and *fta3* mutant. Traits measured are node of flower initiation (NFI), node of leaf change to four leaflets (LC4) and total leaflets around flowering (TL_NFI). (b) Comparison of WT (*ELF3/HR*), WT (5839/*elf3a*), *fta1*, and *lf* (*tfl1c*) genotypes for the same traits. Error bars represent mean ± 1 SE (n = 6-8). Asterisks (*) indicate significant differences from the respective WT control ($P < 0.05$).

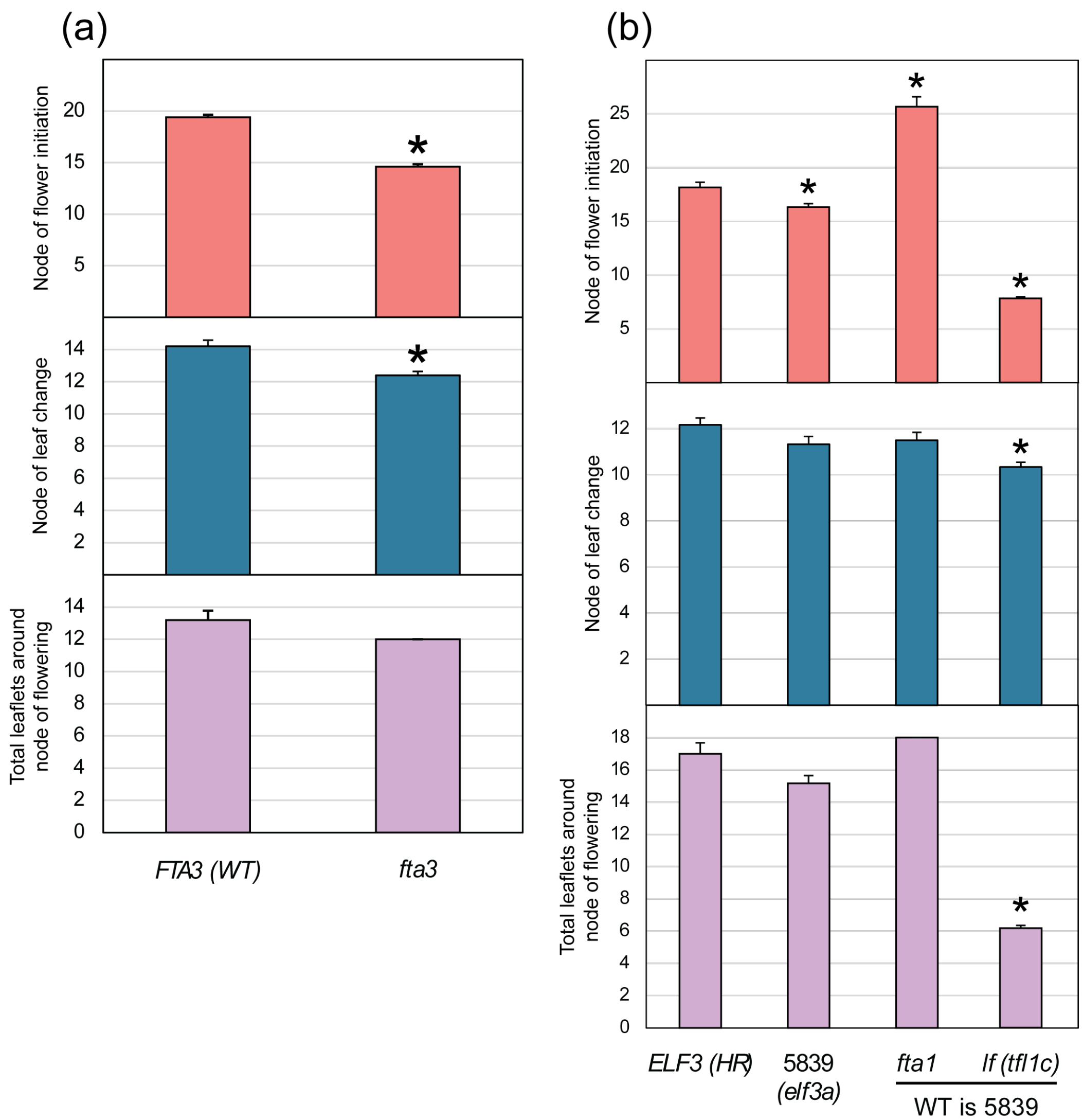

Fig. S3 Identification of *epp1 afila* double mutant phenotype in pea (*Pisum sativum*)
The tendril pattern of the single mutant *afila* and the double mutant *afila epp1* in pea (*Pisum sativum*). Photograph shows the stipules and tendrils of both genotypes excised from node 8. The graph depicts the average number of major tendrils, which refers to the major branches (rachides) that terminate in a tendril(s) rather than all tendrils. Plants were grown in LD conditions. Error bars represent mean ± 1 SE (n = 6). Asterisk (*) denotes statistically significant difference (P<0.05).

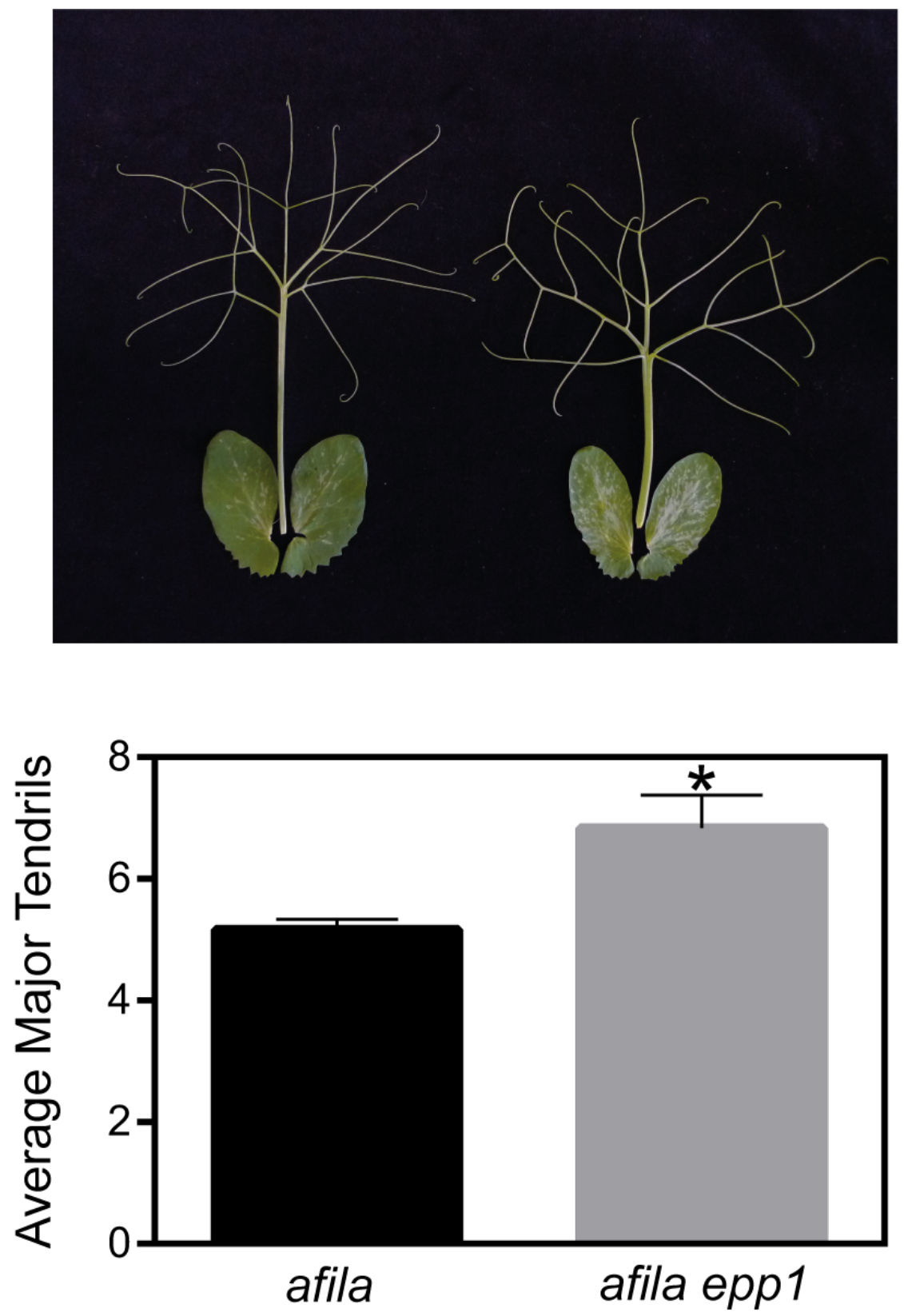

Fig. S4 *A*llelism testing of *epp2* in pea (*Pisum sativum*)
Results of allelism tests between *epp2* mutants and the *epp1* and *aero2* mutants in pea (*Pisum sativum*). (a) Summary of pairwise linkage tests and associated *P* values. *epp2* was not significantly associated with the *epp1* linked marker, JMJ, but did show weak significant linkage with the *aero2* linked marker *TIC22*. (b) Total leaflet number for *epp1* and *epp2* across two JMJ marker classes (a and b) showing significantly fewer leaflets for JMJ *b* genotype compared to *a* genotype in *epp1*, but not *epp2*. Error bars represent mean ± 1 SE. Asterisk (*) denotes significant difference between genotypes.

(a)

Gene pair	P value
epp1 & JMJ	<0.00001
epp2 & JMJ	0.222
aero2 & TIC22	<0.00001
epp2 & TIC22	0.0402

(b)

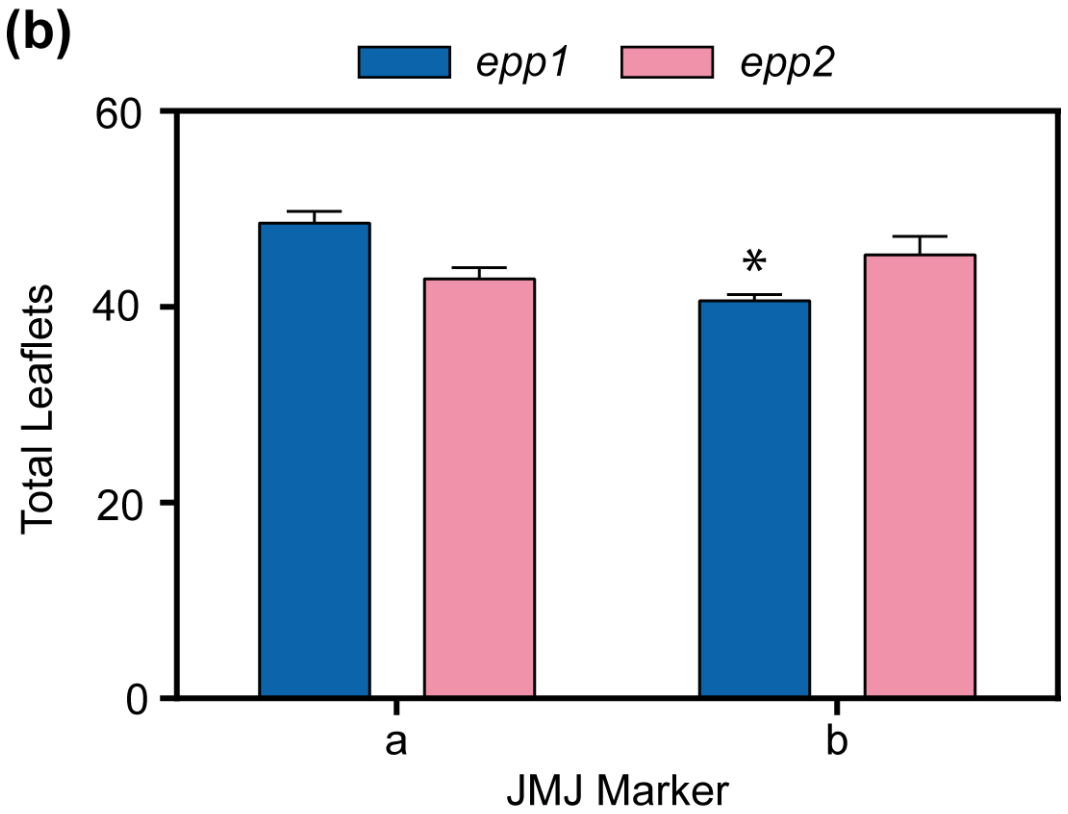

Fig. S5 Phylogenetic relationships of CURLY LEAF family proteins
Maximum-likelihood phylogenetic tree of CLF-related proteins from *Arabidopsis thaliana* (At), chickpea (*Cicer arietinum*, Ca), *Medicago truncatula* (Mt), pea (*Pisum sativum*, Ps), soybean (*Glycine max*, Gm), common bean (*Phaseolus vulgaris*, Pv), maize (*Zea mays*, Zm), rice (*Oryza sativa*, Os), *Brachypodium distachyon* (Bd) and tomato (*Solanum lycopersicum*, Sl). Relevant accession numbers are provided in Table S9. Branch support values are based on 500 bootstrap replicates. CURLY LEAF (CLF) is the *Arabidopsis* orthologue of the *Drosophila* Enhancer of Zeste (E(z)) protein (Goodrich *et al*., 1997). *Arabidopsis* contains three *E(z)*-like genes: *CLF*, *SWINGER* (*SWN*) and *MEDEA* (*MEA*). Legume species exhibit independent duplication events, resulting in four *E(z)*-related genes per diploid genome and eight in tetraploid soybean. Each diploid legume retains a single CLF ortholog, whereas the remaining copies form a distinct legume-specific clade, more closely related to SWN/MEA. Within this clade, the EZL1 subclade is conserved across all legumes, while the EZL2 subclade shows divergence between warm-season phaseoloid legumes and temperate galegoid lineages.

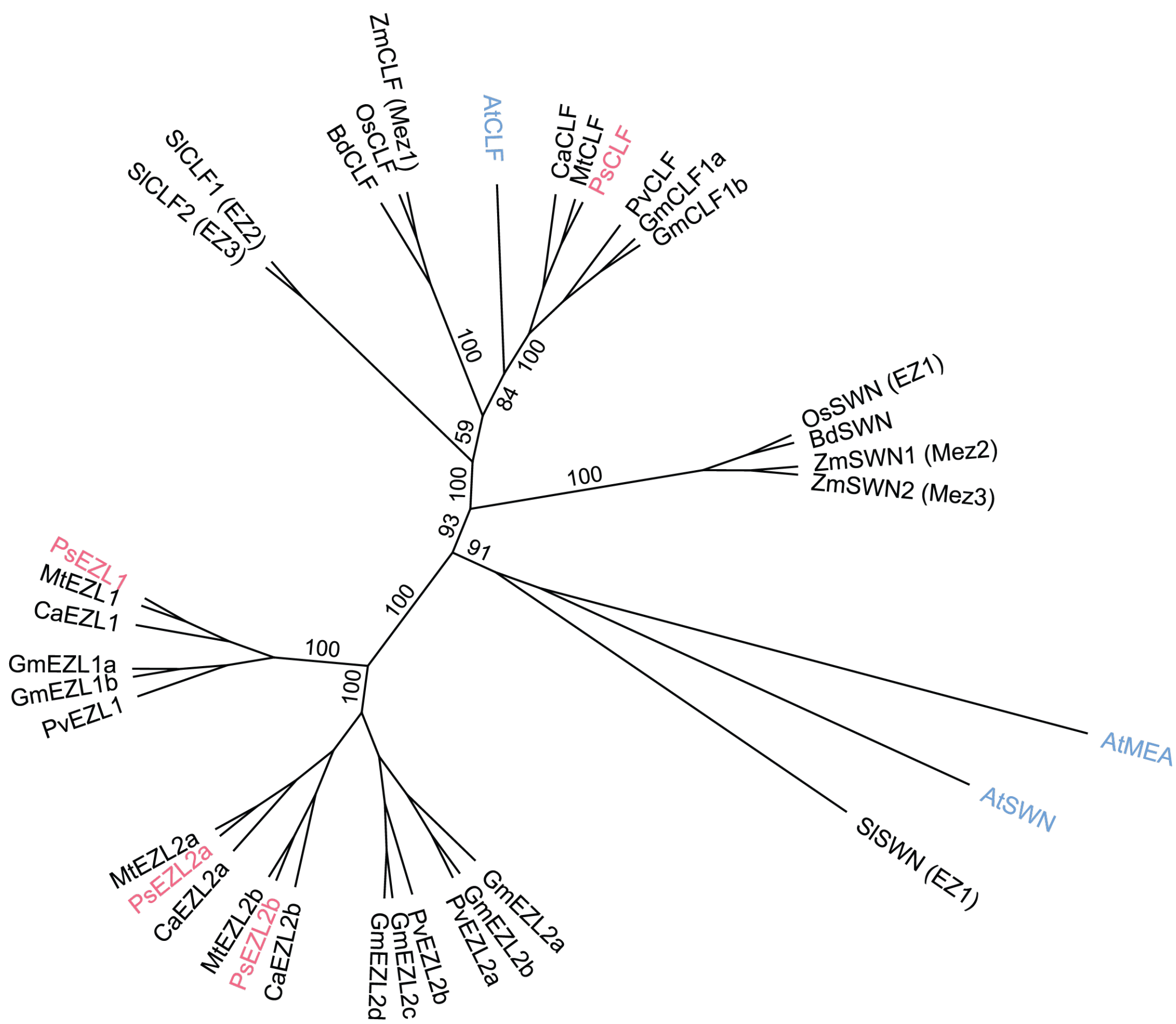

Fig. S6 Phylogenetic relationships of PICKLE family proteins
Maximum-likelihood phylogenetic tree of PKL-related proteins from *Arabidopsis thaliana* (At), chickpea (*Cicer arietinum*, Ca), *Medicago truncatula* (Mt), pea (*Pisum sativum*, Ps), soybean (*Glycine max*, Gm), common bean (*Phaseolus vulgaris*, Pv), maize (*Zea mays*, Zm), rice (*Oryza sativa*, Os), *Brachypodium distachyon* (Bd) and tomato (*Solanum lycopersicum*, Sl). Relevant accession numbers are provided in Table S10. Branch support values are based on 500 bootstrap replicates. Phylogenetic analysis places plant PICKLE (PKL)-related proteins within subfamily II of animal Chromodomain-helicase-DNA-binding (CHD) proteins, indicating shared ancestry and strong conservation (Ho *et al.*, 2013). Across all species examined, including legumes, *PKL*-related genes show high conservation with no evidence of duplication or loss. The distantly related *Arabidopsis SPLAYED* (*SYD*) gene was used as an outgroup to improve resolution. In *Arabidopsis*, two closely related genes, PKL and PKR2, form a clade that includes two paralogous genes from each species, while a second clade contains a single gene from each species orthologous to *Arabidopsis* PKR1.

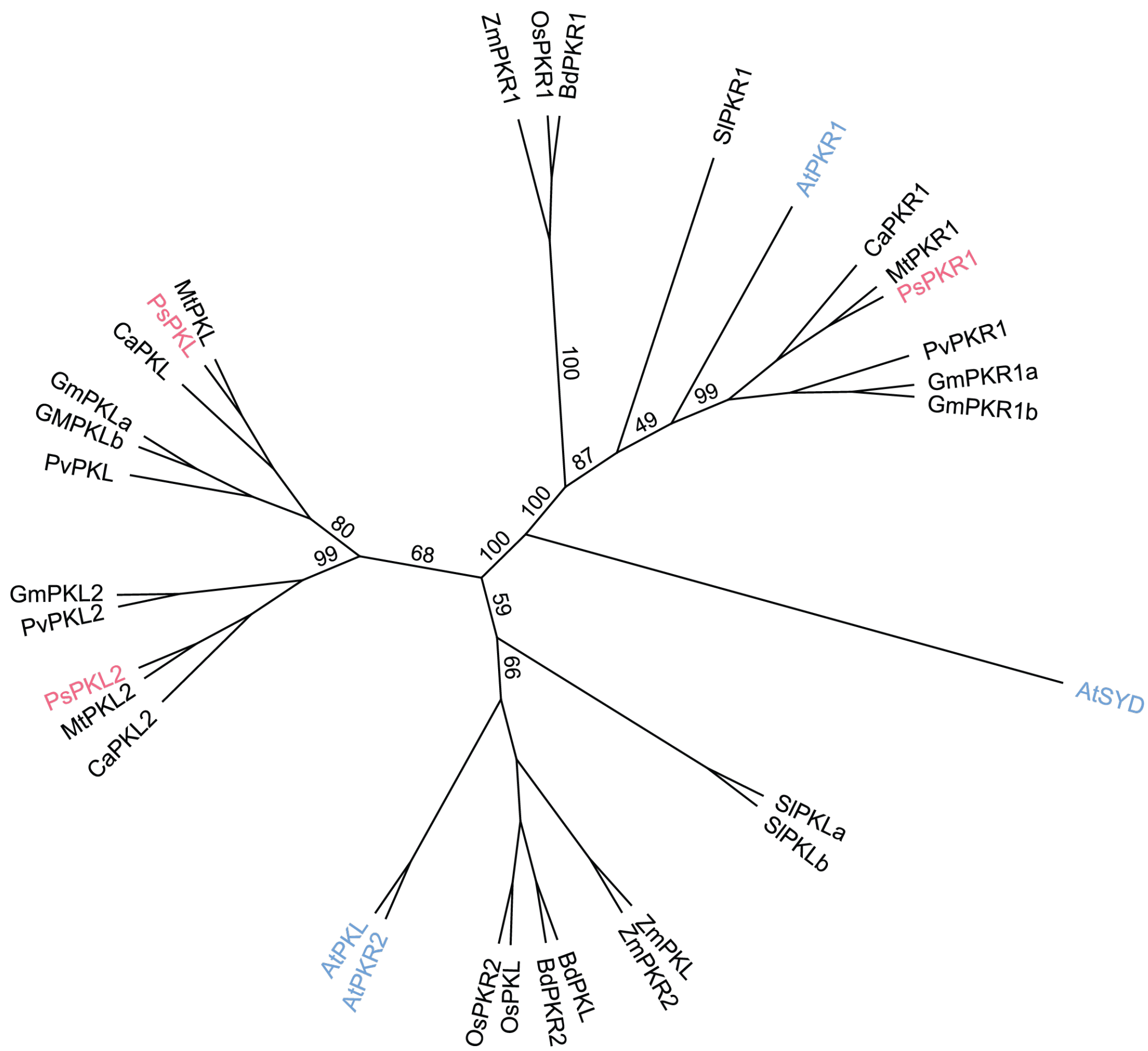

Fig. S7 Phylogenetic relationships of SEUSS family proteins

Maximum-likelihood phylogenetic tree of SEUSS (SEU)-related proteins from *Arabidopsis thaliana* (At), chickpea (*Cicer arietinum*, Ca), *Medicago truncatula* (Mt), pea (*Pisum sativum*, Ps), soybean (*Glycine max*, Gm), common bean (*Phaseolus vulgaris*, Pv), maize (*Zea mays*, Zm), rice (*Oryza sativa*, Os), *Brachypodium distachyon* (Bd) and tomato (*Solanum lycopersicum*, Sl). Relevant accession numbers are provided in Table S11. Branch support values are based on 500 bootstrap replicates. The Arabidopsis *SEU* gene shares sequence similarity with metazoan LIM domain-binding transcriptional adaptors (Franks *et al*., 2002). *Arabidopsis* contains SEU and three SLK genes, which separate into distinct clades. Within the *SLK* clade, duplication events are evident: pea and other cool-season legumes retain a single copy, whereas warm-season legumes, maize and *Arabidopsis* exhibit more recent duplications. In the SEU clade*, Arabidopsis* and tomato each have a single copy, while all other species, including legumes and cereals, show duplications. Among legumes, a single duplication event has occurred, resulting in two paralogous SEU copies.

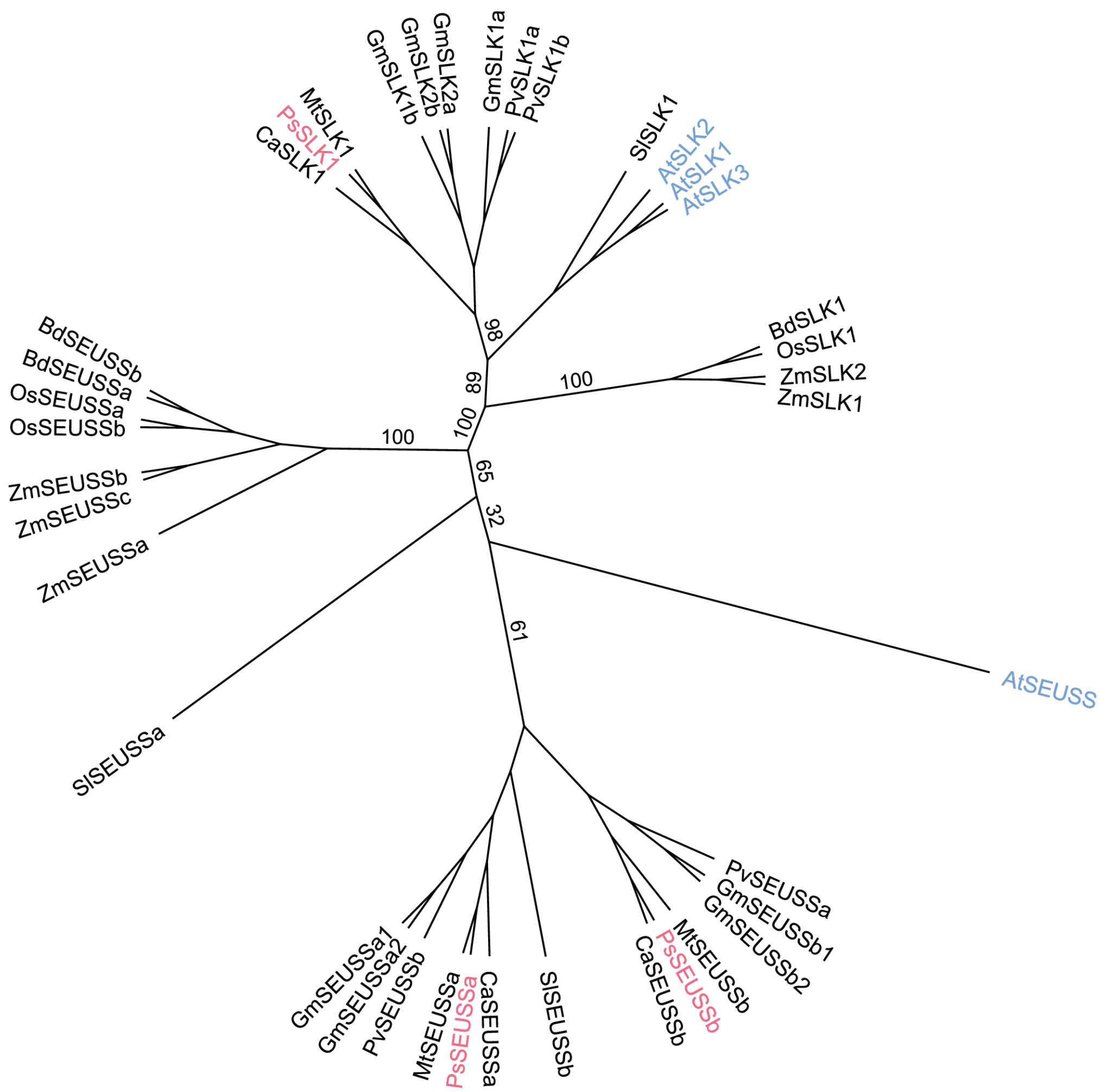

Fig. S8 Characterisation, molecular analysis and allelism testing of the *epp2-2* allele in pea (*Pisum sativum*)

(a) Wild-type (WT; NGB5839) and *epp2-2* plants grown under long-day conditions. Total leaflet number was scored at each developmental node. (b) Putative *SUESSb* candidate gene and the location of the mutation for *epp2-2*. Known functional domains are indicated by black boxes. The region containing the mutation is expanded to show the predicted protein sequence, with the affected amino acid denoted by a black triangle. **(c)** *Epp2-2* allelism testing. The comparison of *epp1* and *epp1* x *epp2-2* F_1 (top panel) and *epp2-1* x *epp2-1* x *epp2-2* F_1 (bottom panel). WT data are shown in both panels to facilitate comparison. Error bars represent mean ± 1 SE (n = 4-6)

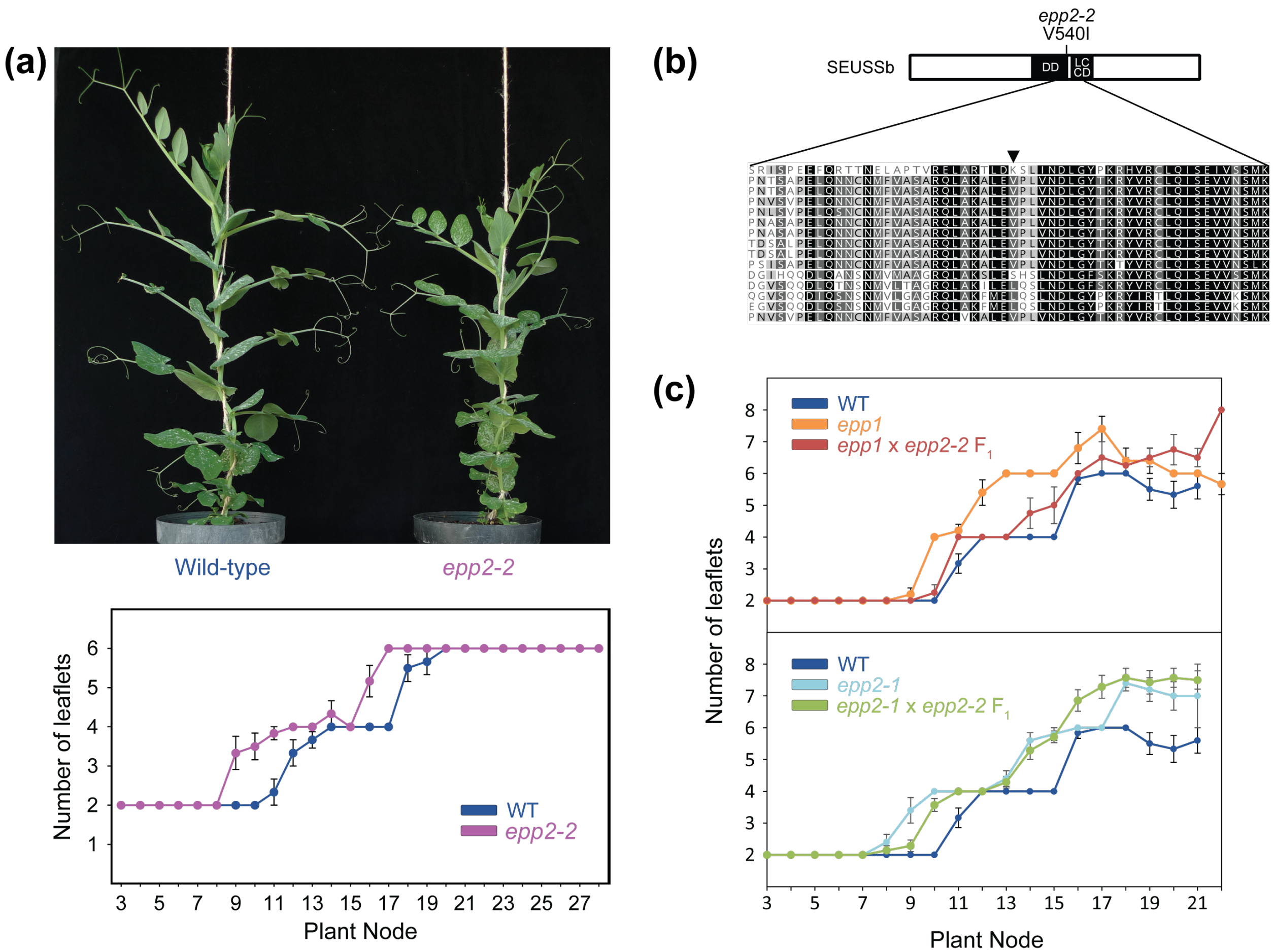

Fig. S9 Expression of candidate and compound leaf development genes in *aero* and *epp* mutants in pea (*Pisum sativum*)

Expression levels were quantified by qRT–PCR from two-week old pea apices (4-6 biological replicates, 2 technical replicates). Transcript abundance was normalized to the geometric mean of *ACTIN* and *TFIIa* and expressed relative to wild-type (WT) levels. Red asterisks indicate significant differences from WT (*, P ≤ 0.05; **, P ≤ 0.01; Welch's t-test). In the boxplots, dots represent individual biological replicates, boxes indicate the interquartile range (IQR; middle 50% of the data), and the centre line indicates the median. The four candidate genes are *CURLYLEAF* (*CLF*), *PICKLE* (*PKL*), *SEUSSa* and *SEUSSb*. The five pea *KNOX1* genes analysed were *STMa*, *STMb*, *KNAT1*, *KNAT2* and *KNAT6*, while *UFO* and *LFY* represent putative downstream targets of KNOX1 activity. Gene identifiers and primer sequences used in this study are provided in Table S8.

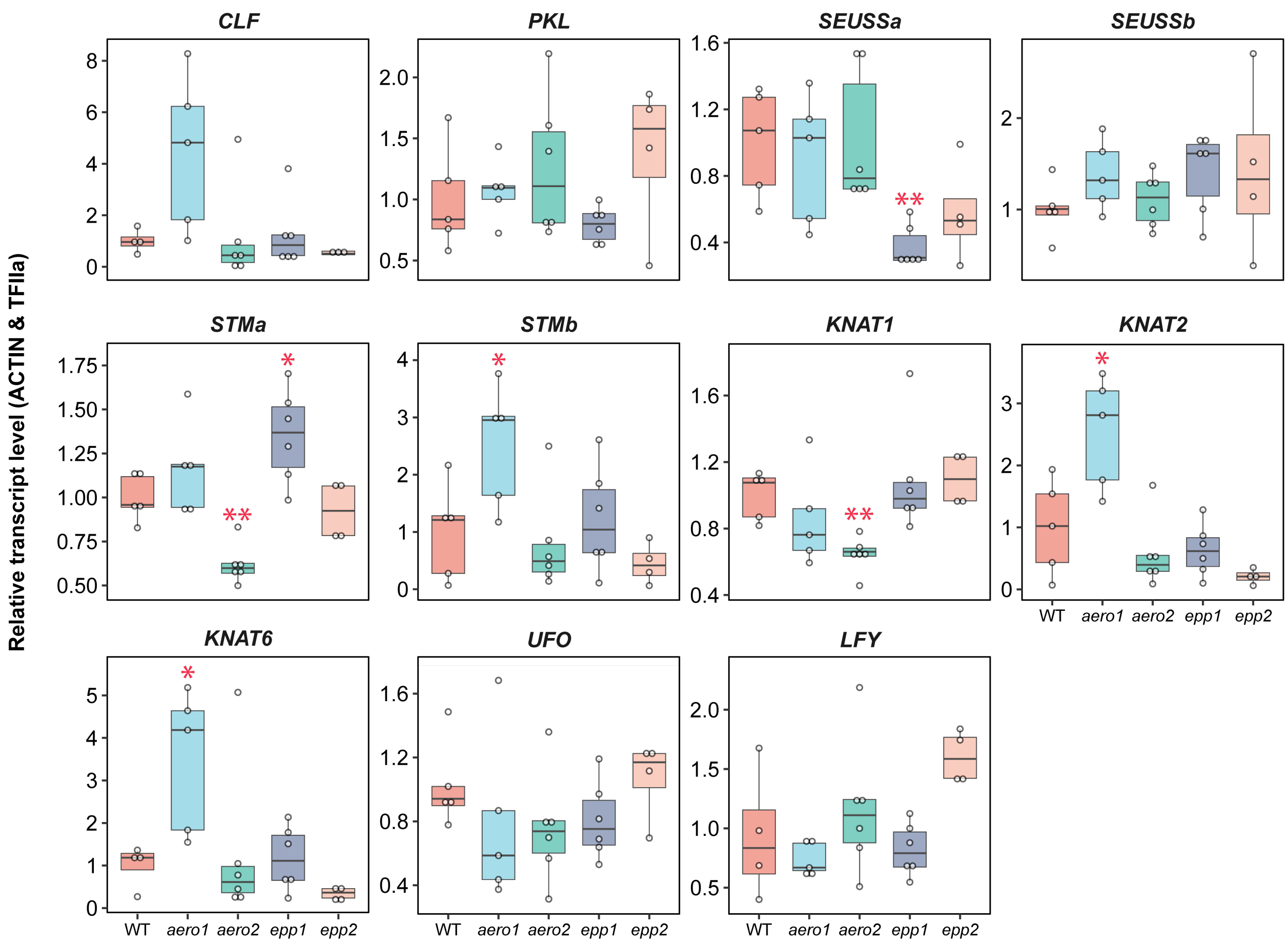

Fig. S10 Pea (*Pisum sativum*) *epp1* and *epp2* inheritance patterns

Histogram data of total leaflets for *epp1* and *epp2* in the (a) F_1 generation and (b) F_2 generation. Controls were also grown at the same time as indicated by the legend colours. Total leaflets from node 3-20 were used for all the F_1 and total leaflets from node 3-18 was used for F_2. The F_2 were grown at a different time and did not produce nodes above 18 consistently across the population.

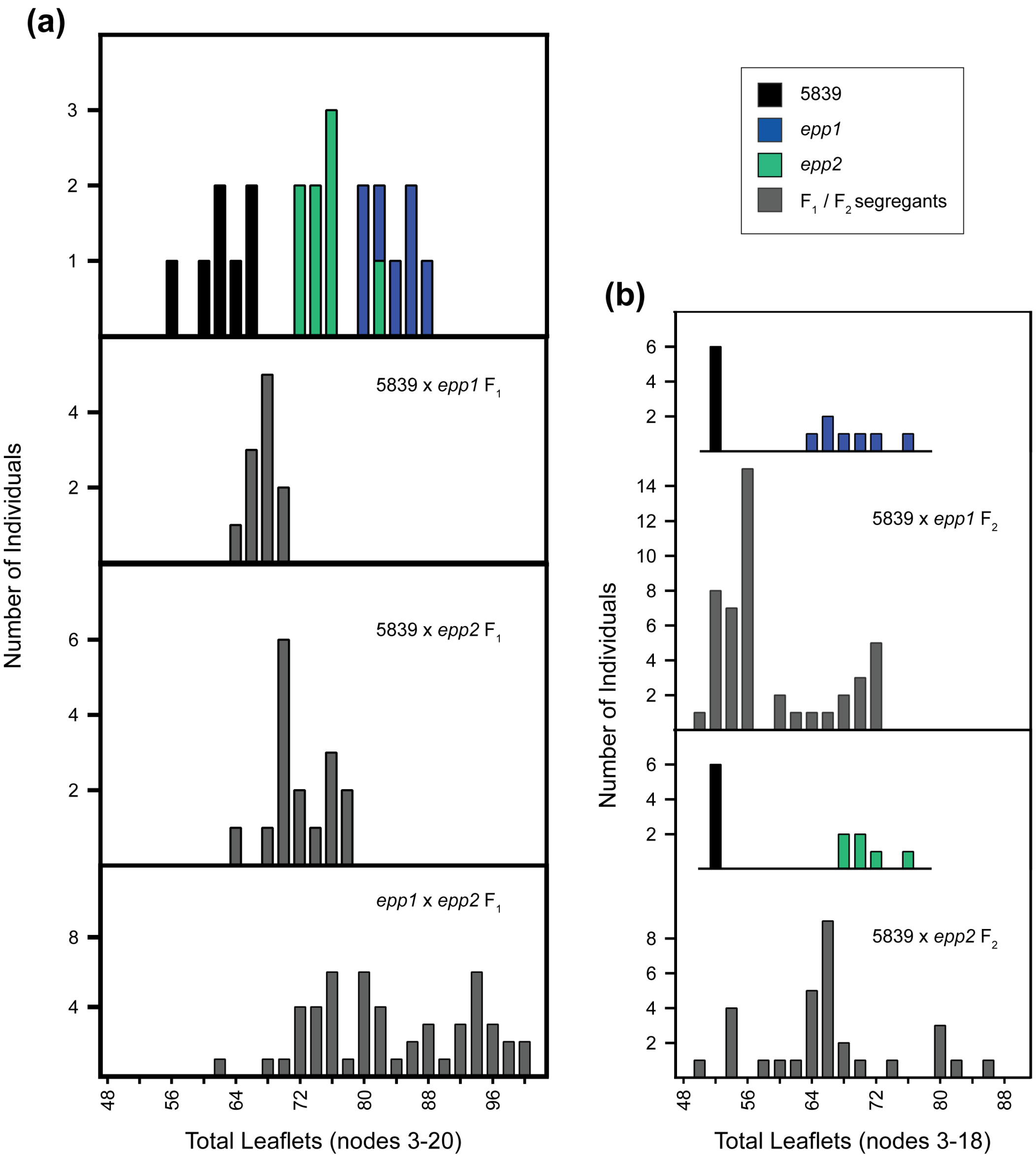

Supporting References

Aubert G, Morin J, Jacquin F, Loridon K, Quillet MC, Petit A, Rameau C, Lejeune-Henaut I, Huguet T, Burstin J. 2006. Functional mapping in pea, as an aid to the candidate gene selection and for investigating synteny with the model legume Medicago truncatula. *Theor Appl Genet* **112**(6): 1024–1041.

Beretta VM, Franchini E, Ud Din I, Lacchini E, Van Den Broeck L, Sozzani R, Orozco-Arroyo G, Caporali E, Adam H, Jouannic S, et al. 2023. The ALOG family members *OsG1L1* and *OsG1L2* regulate inflorescence branching in rice. *The Plant Journal* **115**(2): 351–368.

Blixt S. 1962. Studies in induced mutations in peas VI. *Agri Hortique Genetica* **20**: 95–110.

Bordat A, Savois V, Nicolas M, Salse J, Chauveau A, Bourgeois M, Potier J, Houtin H, Rond C, Murat F, et al. 2011. Translational Genomics in Legumes Allowed Placing In Silico 5460 Unigenes on the Pea Functional Map and Identified Candidate Genes in Pisum sativum L. *G3 (Bethesda)* **1**(2): 93–103.

Caballo C, Berbel A, Ortega R, Gil J, Millán T, Rubio J, Madueño F. 2022. The *SINGLE FLOWER (SFL)* gene encodes a MYB transcription factor that regulates the number of flowers produced by the inflorescence of chickpea. *New Phytologist* **234**(3): 827–836.

Chandler JW, Werr W. 2017. DORNRÖSCHEN, DORNRÖSCHEN-LIKE, and PUCHI redundantly control floral meristem identity and organ initiation in Arabidopsis. *Journal of Experimental Botany* **68**(13): 3457–3472.

Dinneny JR, Weigel D, Yanofsky MF. 2006. *NUBBIN* and *JAGGED* define stamen and carpel shape in *Arabidopsis*. *Development* **133**(9): 1645–1655.

Dixon LE, Pasquariello M, Badgami R, Levin KA, Poschet G, Ng PQ, Orford S, Chayut N, Adamski NM, Brinton J, et al. 2022. MicroRNA-resistant alleles of *HOMEOBOX DOMAIN-2* modify inflorescence branching and increase grain protein content of wheat. *Science Advances* **8**(19).

Fambrini M, Mariotti L, Parlanti S, Salvini M, Pugliesi C. 2015. A GRAS-like gene of sunflower (*Helianthus annuus* L.) alters the gibberellin content and axillary meristem outgrowth in transgenic *Arabidopsis* plants. *Plant Biology* **17**(6): 1123–1134.

Feng G, Qin Z, Yan J, Zhang X, Hu Y. 2011. Arabidopsis *ORGAN SIZE RELATED1* regulates organ growth and final organ size in orchestration with *ARGOS* and *ARL*. *New Phytologist* **191**(3): 635–646.

Franks RG, Wang C, Levin JZ, Liu Z. 2002. *SEUSS*, a member of a novel family of plant regulatory proteins, represses floral homeotic gene expression with *LEUNIG*. *Development* **129**(1): 253–263.

Gómez-Mena C, Sablowski R. 2008. *ARABIDOPSIS THALIANA HOMEOBOX GENE1* Establishes the Basal Boundaries of Shoot Organs and Controls Stem Growth. *The Plant Cell* **20**(8): 2059–2072.

Goodrich J, Puangsomlee P, Martin M, Long D, Meyerowitz EM, Coupland G. 1997. A Polycomb-group gene regulates homeotic gene expression in *Arabidopsis*. *Nature* **386**(6620): 44–51.

Gourlay CW, Hofer JMI, Ellis THN. 2000. Pea Compound Leaf Architecture Is Regulated by Interactions among the Genes *UNIFOLIATA*, *COCHLEATA*, *AFILA*, and *TENDRIL-LESS*. *The Plant Cell* **12**(8): 1279–1294.

Gregis V, Andrés F, Sessa A, Guerra RF, Simonini S, Mateos JL, Torti S, Zambelli F, Prazzoli GM, Bjerkan KN, et al. 2013. Identification of pathways directly regulated by SHORT VEGETATIVE PHASE during vegetative and reproductive development in Arabidopsis. *Genome Biology* **14**(6): R56.

Grigg SP, Canales C, Hay A, Tsiantis M. 2005. SERRATE coordinates shoot meristem function and leaf axial patterning in Arabidopsis. *Nature* **437**(7061): 1022–1026.

Han H, Geng Y, Guo L, Yan A, Meyerowitz EM, Liu X, Zhou Y. 2020. The Overlapping and Distinct Roles of HAM Family Genes in Arabidopsis Shoot Meristems. *Frontiers in Plant Science* **11**.

Hasson A, Plessis A, Blein T, Adroher B, Grigg S, Tsiantis M, Boudaoud A, Damerval C, Laufs P. 2011. Evolution and Diverse Roles of the *CUP-SHAPED COTYLEDON* Genes in *Arabidopsis* Leaf Development. *The Plant Cell* **23**(1): 54–68.

Hecht V, Laurie RE, Vander Schoor JK, Ridge S, Knowles CL, Liew LC, Sussmilch FC, Murfet IC, Macknight RC, Weller JL. 2011. The pea GIGAS gene is a FLOWERING LOCUS T homolog necessary for graft-transmissible specification of flowering but not for responsiveness to photoperiod. *Plant Cell* **23**(1): 147–161.

Ho KK, Zhang H, Golden BL, Ogas J. 2013. PICKLE is a CHD subfamily II ATP-dependent chromatin remodeling factor. *Biochimica et Biophysica Acta (BBA) - Gene Regulatory Mechanisms* **1829**(2): 199–210.

Hofer J, Turner L, Moreau C, Ambrose M, Isaac P, Butcher S, Weller J, Dupin A, Dalmais M, Le Signor C, et al. 2009. *Tendril-less* Regulates Tendril Formation in Pea Leaves. *The Plant Cell* **21**(2): 420–428.

Imlay EE, Jeon H-W, Byrne ME. 2025. Homeodomain transcription factor regulation of leaf serrations in *Arabidopsis thaliana*. *Journal of Experimental Botany* **76**(14): 4014–4026.

Kim S, Soltis PS, Wall K, Soltis DE. 2006. Phylogeny and Domain Evolution in the APETALA2-like Gene Family. *Molecular Biology and Evolution* **23**(1): 107–120.

Lester DR, Mackenzie-Hose AK, Davies PJ, Ross JJ, Reid* JB. 1999. The influence of the null le-2 mutation on gibberellin levels in developing pea seeds. *Plant Growth Regulation* **27**(2): 83–89.

Li M, Zheng Y, Cui D, Du Y, Zhang D, Sun W, Du H, Zhang Z. 2022. GIF1 controls ear inflorescence architecture and floral development by regulating key genes in hormone biosynthesis and meristem determinacy in maize. *BMC Plant Biology* **22**(1).

Li S. 2015. The *Arabidopsis thaliana* TCP transcription factors: A broadening horizon beyond development. *Plant Signaling & Behavior* **10**(7): e1044192.

Liu Y, Yang Y, Wang R, Liu M, Ji X, He Y, Zhao B, Li W, Mo X, Zhang X, et al. 2023. Control of compound leaf patterning by MULTI-PINNATE LEAF1 (MPL1) in chickpea. *Nature Communications* **14**(1).

Lozano-Sotomayor P, Chávez Montes RA, Silvestre-Vañó M, Herrera-Ubaldo H, Greco R, Pablo-Villa J, Galliani BM, Diaz-Ramirez D, Weemen M, Boutilier K, et al. 2016. Altered expression of the bZIP transcription factor DRINK ME affects growth and reproductive development in *Arabidopsis thaliana*. *The Plant Journal* **88**(3): 437–451.

Lu Z, Zhang J, Wang H, Zhang K, Gu Z, Xu Y, Zhang J, Wang M, Han L, Xiang F, et al. 2024. Rewiring of a KNOXI regulatory network mediated by UFO underlies the compound leaf development in Medicago truncatula. *Nature Communications* **15**(2988).

Lv Z, Zhao W, Kong S, Li L, Lin S. 2023. Overview of molecular mechanisms of plant leaf development: a systematic review. *Frontiers in Plant Science* **14**.

Maier AT, Stehling-Sun S, Offenburger S-L, Lohmann JU. 2011. The bZIP Transcription Factor PERIANTHIA: A Multifunctional Hub for Meristem Control. *Frontiers in Plant Science* **2**.

Marx G. 1986. Linkage of an aeromaculata mutant on chromosome 1. *Pisum Newsletter* **18**: 42–44.

Mo X, He L, Liu Y, Wang D, Zhao B, Chen J. 2022. The Genetic Control of the Compound Leaf Patterning in Medicago truncatula. *Frontiers in Plant Science* **12**(749989).

Mohammed B, Bilooei SF, Dóczi R, Grove E, Railo S, Palme K, Ditengou FA, Bögre L, López-Juez E. 2018. Converging Light, Energy and Hormonal Signaling Control Meristem Activity, Leaf Initiation, and Growth. *Plant Physiology* **176**(2): 1365–1381.

Murfet I, Taylor S. 2004. The aero2 (aeromaculata2) mutation in pea increases leaf flecking and complexity but, unlike aero1, does not promote flowering. *Pisum Genetics* **36**: 14–19.

Peaucelle A, Louvet R, Johansen JN, Salsac F, Morin H, Fournet F, Belcram K, Gillet F, Höfte H, Laufs P, et al. 2011. The transcription factor BELLRINGER modulates phyllotaxis by regulating the expression of a pectin methylesterase in *Arabidopsis*. *Development* **138**(21): 4733–4741.

Refli. 2002. *The physiological and genetic control of vegetative phase change in Pisum sativum L.* MSc thesis, University of Tasmania Hobart, Tasmania.

Sessa G, Carabelli M, Sassi M. 2024. The Ins and Outs of Homeodomain-Leucine Zipper/Hormone Networks in the Regulation of Plant Development. *International Journal of Molecular Sciences* **25**(11): 5657.

Sidorova K, Uzhintseva L. 1995. Analysis of induced supaeromaculata mutants in pea. *Genetika (Russian Federation)* **31**(8): 1177–1179.

Stahle MI, Kuehlich J, Staron L, Von Arnim AG, Golz JF. 2009. YABBYs and the Transcriptional Corepressors LEUNIG and LEUNIG_HOMOLOG Maintain Leaf Polarity and Meristem Activity in *Arabidopsis*. *The Plant Cell* **21**(10): 3105–3118.

Sussmilch FC, Berbel A, Hecht V, Vander Schoor JK, Ferrándiz C, Madueño F, Weller JL. 2015. Pea VEGETATIVE2 Is an FD Homolog That Is Essential for Flowering and Compound Inflorescence Development. *The Plant Cell* **27**(4): 1046–1060.

Tattersall AD, Turner L, Knox MR, Ambrose MJ, Ellis THN, Hofer JMI. 2005. The Mutant *crispa* Reveals Multiple Roles for *PHANTASTICA* in Pea Compound Leaf Development. *The Plant Cell* **17**(4): 1046–1060.

Tayeh N, Hofer JMI, Aubert G, Jacquin F, Turner L, Kreplak J, Paajanen P, Le Signor C, Dalmais M, Pflieger S, et al. 2024. *afila*, the origin and nature of a major innovation in the history of pea breeding. *New Phytologist* **243**(3): 1247–1261.

Taylor SA, Murfet IC. 2003. A supaeromaculata mutation affects heterochrony in pea. *Physiologia Plantarum* **117**(1): 100–107.

Vercruyssen L, Verkest A, Gonzalez N, Heyndrickx KS, Eeckhout D, Han S-K, Jégu T, Archacki R, Van Leene J, Andriankaja M, et al. 2014. ANGUSTIFOLIA3 Binds to SWI/SNF Chromatin Remodeling Complexes to Regulate Transcription during Arabidopsis Leaf Development. *The Plant Cell* **26**(1): 210–229.

Vlad D, Kierzkowski D, Rast MI, Vuolo F, Dello Ioio R, Galinha C, Gan X, Hajheidari M, Hay A, Smith RS, et al. 2014. Leaf Shape Evolution Through Duplication, Regulatory Diversification, and Loss of a Homeobox Gene. *Science* **343**(6172): 780–783.

Wang D, Zhao B, Zhou X, Zhou S, Yang L, Mao Y, Bai Q, Zhao W, Sun M, Liu M, et al. 2025. Synergistic effects of *GmLFYa* and *GmLFYb* on Compound Leaf Development in Soybean. *Physiologia Plantarum* **177**(1).

Wang H, Kong F, Zhou C. 2021. From genes to networks: The genetic control of leaf development. *Journal of Integrative Plant Biology* **63**(7): 1181–1196.

Wang Y, Jiao Y. 2023. Cell signaling in the shoot apical meristem. *Plant Physiology* **193**(1): 70–82.

Weeden NF, Coyne CJ, Lavin M, McPhee K. 2021. Distinguishing among Pisum accessions using a hypervariable intron within Mendel's green/yellow cotyledon gene. *Genetic Resources and Crop Evolution* **68**(6): 2591–2609.

Weller JL, Hecht V, Liew LC, Sussmilch FC, Wenden B, Knowles CL, Vander Schoor JK. 2009. Update on the genetic control of flowering in garden pea. *Journal of Experimental Botany* **60**(9): 2493–2499.

Weller JL, Liew LC, Hecht VFG, Rajandran V, Laurie RE, Ridge S, Wenden B, Vander Schoor JK, Jaminon O, Blassiau C, et al. 2012. A conserved molecular basis for photoperiod adaptation in two temperate legumes. *Proceedings of the National Academy of Sciences* **109**(51): 21158–21163.

Weller JL, Murfet IC, Reid JB. 1997. Pea Mutants with Reduced Sensitivity to Far-Red Light Define an Important Role for Phytochrome A in Day-Length Detection. *Plant Physiology* **114**(4): 1225–1236.

Williams O, Vander Schoor JK, Butler JB, Ridge S, Sussmilch FC, Hecht VFG, Weller JL. 2022. The genetic architecture of flowering time changes in pea from wild to crop. *Journal of Experimental Botany* **73**(12): 3978–3990.

Xu Y, Qian Z, Zhou B, Wu G. 2019. Age-dependent heteroblastic development of leaf hairs in *Arabidopsis*. *New Phytologist* **224**(2): 741–748.

Yao WJ, Wang YP, Peng J, Yin PP, Gao H, Xu L, Laux T, Zhang XS, Su YH. 2024. The RPT2a–MET1 axis regulates *TERMINAL FLOWER1* to control inflorescence meristem indeterminacy in Arabidopsis. *The Plant Cell* **36**(5): 1718–1735.

Zahn IE, Roelofsen C, Angenent GC, Bemer M. 2023. TM3 and STM3 Promote Flowering Together with FUL2 and MBP20, but Act Antagonistically in Inflorescence Branching in Tomato. *Plants* **12**(15): 2754.

Zhu W, Yang L, Wu D, Meng Q, Deng X, Huang G, Zhang J, Chen X, Ferrándiz C, Liang W, et al. 2022. Rice *SEPALLATA* genes *OsMADS5* and *OsMADS34* cooperate to limit inflorescence branching by repressing the *TERMINAL FLOWER1*-like gene *RCN4*. *New Phytologist* **233**(4): 1682–1700.
